# Synergistic effect of carbon fabric metastructure and magnetic layers in a composite for electromagnetic radiation shielding

**DOI:** 10.1038/s41598-026-61026-3

**Published:** 2026-07-08

**Authors:** Yevhen Yashchyshyn, Alexander Zaichenko, Khrystyna Harhay, Nataliya Mitina, Konrad Godziszewski, Oleh Izhyk, Ivan Demydov, Roman Nebesnyi, Vyacheslav Khardikov, Vladimir Tuz

**Affiliations:** 1 Institute of Radioelectronics and Multimedia Technology, Warsaw University of Technology, Nowowiejska 15/19, 00-665 Warsaw, Poland; 2 Department of Organic Chemistry, Institute of Chemistry and Chemical Technologies, Lviv Polytechnic National University, 2, Svyatyi Yuriy Square, Lviv, 79013 Ukraine; 3 Department of Optoelectronics and Information Technologies, Faculty of Electronics and Computer Technologies, Ivan Franko National University of Lviv, 107, Generala Tarnavskogo Street, Lviv, 79017 Ukraine; 4 Department of Information and Communication Technologies, Lviv Polytechnic National University, 2, Profesorska Street, Lviv, 79013 Ukraine; 5 Department of Organic Products Technology, Institute of Chemistry and Chemical Technologies, Lviv Polytechnic National University, 2, Svyatyi Yuriy Square, Lviv, 79013 Ukraine; 6 School of Radiophysics, Biomedical Electronics and Computer Systems, V. N. Karazin Kharkiv National University, 4, Svobody Square, Kharkiv, 61022 Ukraine; 7 National Technical University “Kharkiv Polytechnic Institute”, 2, Kyrpychova Street, Kharkiv, 61002 Ukraine

**Keywords:** Carbon fabric, Shielding effectiveness, Electromagnetic shielding materials, Millimeter-wave absorbers, Sub-terahertz range, Magnetic films, Engineering, Materials science, Physics

## Abstract

The widespread use of high-frequency electromagnetic radiation (EMR) in communication, defense, medical, and household technologies raises health and safety concerns, underscoring the demand for efficient shielding and absorption materials. This work presents flexible protective composites based on conductive carbon fabric coated with thin magnetic polymer films. Several multilayer configurations were prepared and characterized, consisting of carbon fabric coated with epoxy polymer layers containing Ni or Fe$_2$O$_3$ fillers. The shielding performance, primarily governed by the fabric’s regular metastructure, exhibits strong polarization dependence in the *90-330* GHz range. The combined interaction of conductive carbon fabric and magneto-responsive polymer layers enhances absorption, induces multiple resonances, and broadens the effective shielding bandwidth, resulting in significantly reduced reflection across a wide spectral range.

## Introduction

Extending the operating range of radars to higher frequencies necessitates the development of advanced composites and coatings capable of absorbing and shielding electromagnetic radiation (EMR) to protect both airborne and ground-based targets^1^. Protecting humans from EMR emitted by high-frequency radio engineering systems, mitigating radio-frequency pollution from electronic and telecommunication devices, countering electromagnetic weapons, ensuring airport security, and enabling medical imaging further emphasize the urgency of designing effective EMR-protective materials^2,3^. In recent years, intensive research has been devoted to developing composites for shielding electromagnetic radiation in the sub-terahertz frequency range for multifunctional applications^4,5^.

Systematic studies have shown that EMR absorbers typically consist of a matrix based on thermosetting polymers, thermoplastics, or their blends, and combined with fillers of defined size and composition^6–12^. Common fillers include conductive or semiconductive materials (e.g., carbon, soot, graphene, graphene oxide), materials with high permittivity (e.g., oxides of Fe, Ni, Zn, Sn), ferro- or ferrimagnetic particles with strong penetration capability to combine both magnetic and dielectric properties^13,14^. Traditional absorbers are typically fabricated using low-loss backings, such as silicone rubber, incorporating magnetic or dielectric foams and resins. The inclusion of magnetic materials has been shown to reduce coating thickness while broadening the absorption band.

In absorbers composed of a resistive layer on a metallic backing, the input impedance is determined by the surface resistance of the resistive layer, positioned at approximately one-quarter wavelength from the ground plane^15^. The resonant frequency of the coating configuration constrains the absorption band of such systems. Stepped absorber coatings, consisting of multiple resistive layers separated by quarter-wavelength distances, are particularly effective. Their gradual increase in resistance with distance from the ground plane allows electromagnetic waves to penetrate progressively deeper, minimizing reflection in the outer layers and enabling broadband absorption. However, while increasing the number of layers broadens the transmission band, it also reduces mechanical strength. Protective composite coatings containing dielectric, conductive, and magnetic particles such as graphite, iron, ferrites, silicon carbide, molybdenum disulfide, and spinels remain promising candidates for advanced absorbers^16,17^.

In recent years, extensive efforts have been devoted to developing lightweight and flexible electromagnetic shielding composites by incorporating conductive fillers (e.g., carbon nanotubes, graphene, MXenes) and magnetic components into polymer matrices, as well as by constructing multilayer or hierarchical architectures to enhance absorption performance^18–24^. These studies demonstrate the effectiveness of combining conductive and magnetic losses and optimizing structural design for EMI shielding, primarily in the MHz-GHz frequency range. However, experimental investigations targeting absorption-dominated shielding at millimeter-wave and sub-terahertz frequencies remain limited, and the role of intrinsic substructures in coupling with magnetic coatings to tailor high-frequency electromagnetic responses is still insufficiently explored.

Among emerging systems, thermoplastic matrices incorporating quasi-one-dimensional transition-metal trichalcogenides^25^, as well as porous composites based on carbon powder and tetraethoxysilane^26^, have drawn increasing interest. Carbon-based materials are especially attractive as EMR absorbers owing to their strong EMR-to-heat conversion, low cost, excellent chemical and thermal stability, high permittivity, and superior conductivity^27^. For instance, lightweight 3D aerogels (*2.0-8.0* mg/cm$^3$) fabricated from graphene and alkaline lignin via sublimation have demonstrated shielding efficiency of *21.3-49.2* dB^28^. However, the high conductivity of carbon materials often degrades impedance matching, limiting microwave absorption^29^. To overcome this limitation, graphene-based absorbers are typically combined with low-dielectric ($\epsilon _r < 5$) or magnetic components (e.g., polymers, SiO$_2$, NiO, *α*-Fe$_2$O$_3$), which reduce the composite’s relative permittivity ($\epsilon _r \sim 10-20$) and improve impedance matching^30–32^. Incorporating magnetic fillers (Fe, Co, Ni, ferrites) either within carbon particles or as surface coatings further enhances performance by reducing surface reflection and promoting magnetic loss mechanisms^33^.

Flexible sandwich-structured films based on polyester nonwoven fabric and thermoplastic polyurethane with *∼ 20* % graphene have demonstrated not only excellent flexibility and durability, but also shielding efficiency ($\text {SE} \ge 20$ dB) up to 49.1 GHz across the *5.4-59.6* GHz frequency range, at a thickness of only $\sim 50~\mu$m^34^. Woven and nonwoven reinforcements are also attractive for EMR-protective coatings because of their porous structure, controllable thickness, and low cost^35,36^. In these materials, pore size and internal structure strongly influence EMR loss via multiple resonances, enhancing shielding efficiency. Nonwoven-based composites, with their high porosity, large surface area, and strong interfacial interactions, are particularly effective for EMR absorption and scattering. For example, polysiloxane-fiber composites have achieved shielding efficiencies of *50-56* dB in the *8.2-26.5* GHz range^37^.

Advanced fabrication techniques, such as electrospinning, enable layer-by-layer construction of reinforced composites with *in situ* polymerization^38^. Conductive shielding composites can also be produced through surface activation followed by deposition of conductive polymer/inorganic layers^39^, by mixing with conductive fibers^40^, or by incorporating conductive and magnetic particles^41^. Materials with periodic arrangements of conductive or magnetic units (metamaterials) are another promising class^42^. Typically consisting of metallic patterns on dielectric substrates, they exhibit frequency-selective absorption and, in some cases, negative permittivity and/or permeability. Broadband absorption in monolayer metamaterial absorbers is pursued either by designing multi-frequency unit cells or by dynamically tuning electromagnetic parameters to shift resonant peaks. However, monolayer metamaterials rarely achieve true broadband absorption, motivating hybrid designs that combine conventional absorbers with metamaterials^43^. Such hybrid systems leverage multiple resonances in stratified coatings, significantly enhancing overall absorption.

Resonant phenomena play a central role in EMR shielding^44^. Resonances arise when structural, electrical, or magnetic features of a material align with the frequency of incoming radiation, leading to maximal energy transfer. Depending on design, resonance may enhance EMR absorption, scattering, or reflection, enabling applications ranging from radar stealth and signal blocking to medical shielding. Different types of resonances are exploited^45^:

- Dielectric resonances, which occur when polar molecules or dipoles oscillate in phase with the incident field (e.g., BaTiO$_3$- or SiC-filled polymers). Attenuation arises from interfacial, dipolar, defect-induced polarization, and conductive loss.
- Magnetic resonances, which occur when EMR frequencies match the precession of magnetic dipoles in fillers such as Fe$_3$O$_4$, Co, Ni, or spinel ferrites. Energy is dissipated through spin-lattice and spin-spin relaxation. Spinel ferrites (MFe$_2$O$_4$, M = Fe, Co, Ni, Zn) are especially effective because they combine dielectric and magnetic losses, tunable via cation distribution.
- Structural (geometric) resonances, which arise from periodic features, layer thickness, or core-shell geometries that act as resonant cavities or antennas. Porous, multilayered, and 3D composites enhance absorption through multiple reflections and scattering.

By integrating materials with complementary electromagnetic properties (e.g., magnetic layers, conductive grids, and carbon nanotubes), it is possible to minimize reflection while maximizing absorption across multiple frequency bands. Such hybrid architectures enable precise tuning of resonant frequencies while optimizing shielding performance. Metamaterial-based absorbers, in particular, offer unique advantages over traditional absorbers, including miniaturization, tunability, and enhanced efficiency. Their performance depends on composition, microstructure, and periodicity, which together facilitate coupled dielectric, magnetic, and geometric interactions, paving the way for next-generation ultra-wideband EMR shielding materials. Building upon these insights, the present study introduces a flexible, multilayer carbon fabric-based composite that combines the geometric advantages of a woven metastructure with magnetic and conductive coatings for sub-terahertz EMR shielding.

In support of this methodology, this paper reports high microwave EMR absorption and shielding efficiency in a newly developed flexible carbon fabric-based composite. We attribute this performance to electromagnetic resonance effects arising from the coupled interaction between the anisotropic periodic metastructure of the woven carbon fabric and the magneto-dielectric response of the magnetic polymer/inorganic surface layers. The observed enhancement is interpreted as a combined effect of conductive, dielectric, magnetic, and geometric contributions that improve impedance matching, promote multiple resonances, and suppress reflection over a broad spectral range.

## Materials and methods

### Materials

The epoxy resin Epidian 57 (bisphenol-A-based; molecular weight 340 Da; epoxy group content 23.8 %) was supplied by HCIECH Sarzyna (Nowa Sarzyna, Poland). Polyethylene polyamine (PEPA), purchased from Silkor (Kyiv, Ukraine), was used as the curing agent for the epoxy resin. Dioctyl phthalate (DOP) was obtained from HLR (Kyiv, Ukraine) and used as the plasticizer. Graphite nanoplatelets (GNP6, RMC Remacon GmbH, Bad Säckingen, Germany) had a particle size of *3.5-6.0* *μ*m and a carbon content of 99.5 %. Nickel carbonyl powder (Ukrstarline, Nikopol, Ukraine) exhibited a particle size *>10* *μ*m, Ni content of 99.9 %, and a face-centered cubic (FCC) crystal structure. Gamma iron oxide (*γ*-Fe$_2$O$_3$) micropowder (Nanografi Advanced Materials, Jena, Germany) had a particle size of *1-3* *μ*m, purity of 99.9 %, and an FCC crystal structure. Carbon fabric (12K, 480 tex; SkyCarbon, Kharkiv, Ukraine) has a thickness of 0.52 mm, an areal density of 400 g/m$^2$, tensile strength of 4480 MPa, and Young’s modulus of *220-245* GPa.

### Samples preparation

**Fig. 1:**
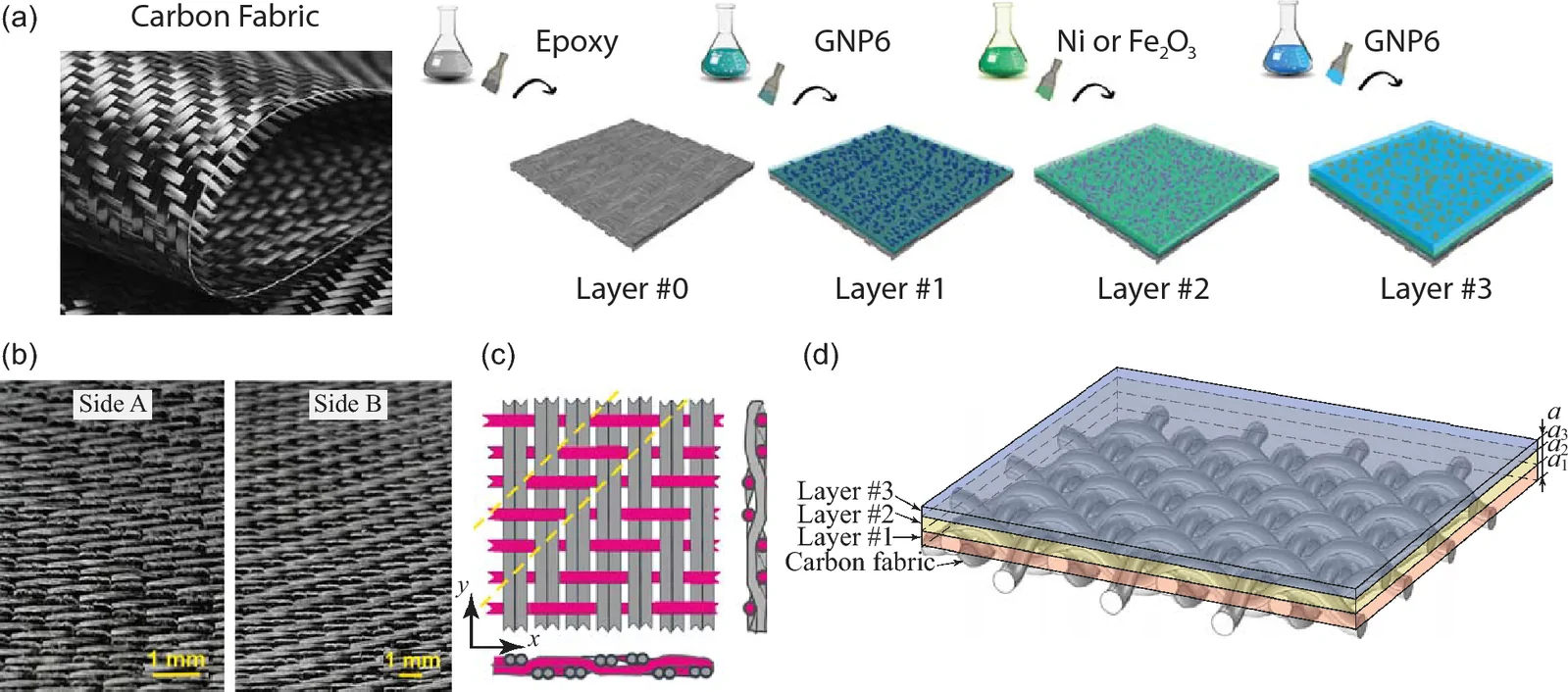
Structure of the shielding composite based on conductive carbon fabric (**a**) Scheme of the preparation procedure of a three-layer configuration, in which the fabric substrate is sequentially coated with epoxy-based films; Layer #2 contains magnetic particles (Ni or Fe$_2$O$_3$) that provide magnetic loss. (**b**) Photographs of the front (Side A) and back (Side B) surfaces of the carbon fabric, where the scale bars are 1 mm. (**c**) Schematic of the twill (diagonal) weave, showing the distribution of warp and weft threads and the resulting oblique bundle alignment. (**d**) Schematic diagram of the final shielding composite. The fabric substrate has a thickness of $a_0 = 1.0$ mm, and successive coatings increase the total thickness *a* to *1.3-1.8* mm depending on the sample.

The multilayer architecture and material compositions were selected based on a functionally graded electromagnetic design concept, in which each constituent layer performs a distinct and complementary role in the overall shielding response. The woven carbon fabric serves as a mechanically robust and electrically conductive substrate with an intrinsic anisotropic metastructure, enabling polarization-dependent geometric resonances. The polymer coating layers were then designed to progressively tailor impedance matching and loss mechanisms across the thickness of the composite. The preparation scheme of the composite is presented in Fig. 1(a).

Carbon fabric was selected as the substrate for the composite EMR absorber owing to its inherent electrical conductivity and radar-absorbing properties. An epoxy resin was employed as the matrix, modified with dioctyl phthalate (plasticizer) and polyethylene polyamine (PEPA, curing agent) in a mass ratio of 10:1:1 (resin/plasticizer/hardener). The initial thickness of the carbon fabric substrate was $a_0 = 1.0$ mm.

Graphite was selected as a conductive filler in the outer polymer layers due to its moderate conductivity and broadband dielectric loss, which enhances ohmic dissipation while avoiding severe impedance mismatch associated with highly conductive fillers. Nickel and iron(III) oxide were chosen as representative magnetic fillers with distinct magnetic permeability dispersion, enabling the introduction of magnetic loss and resonance broadening without significantly increasing material density or rigidity. The selected filler concentrations were sufficient to activate dielectric and magnetic losses while preserving coating homogeneity, flexibility, and adhesion to the carbon fabric substrate.

Therefore, the absorber was fabricated as a multilayer composite using a sequential coating process. To evaluate the contribution of each structural component, three main samples were prepared: Sample #1 with a single coating layer, Sample #2 with two coating layers, and Sample #3 with three coating layers. Additionally, two auxiliary configurations (designated as Sample #2$^\prime$ and Sample #3$^\prime$) were fabricated. These auxiliary structures mimic the geometry and layer composition of their respective main counterparts (Samples #2 and #3) but completely omit Layer #1 to isolate and precisely evaluate its electromagnetic contribution.

The base polymer composite layer (Layer #1, $a_1 = 0.3$ mm) was prepared by dispersing graphite (6 wt %) into the resin mixture, homogenizing by manual stirring for 5 min, coating the carbon fabric uniformly, and curing at $20~^\circ$C for 15 hours. This configuration is designated as Sample #1.

Subsequently, a second coating (Layer #2, $a_2 = 0.3$ mm) was applied. For Sample #2, nickel powder (32 wt %) was incorporated into the resin, whereas for Sample #3, iron(III) oxide (23 wt %) was used as the filler. Each mixture was stirred for 5 minutes, applied to the cured base layer, and cured under identical conditions ($20~^\circ$C, 15 hours).

Finally, Sample #3 received a third layer (Layer #3, $a_3 = 0.2$ mm) containing graphite (6 wt %). This layer was prepared, applied, and cured following the same procedure as the previous layers. The total thickness of the sample is $a=\sum _{j=0}^3 a_j$.

The carbon fabric used as the substrate has a twill (diagonal) weave. Unlike a plain rectangular weave, in which warp and weft threads alternate directly over and under each other, the twill structure introduces a lateral shift between successive rows. Each weft thread passes over multiple warp threads before going under the next ones, and the pattern is progressively offset in adjacent rows. This produces a diagonal orientation of the bundles and a repeating oblique stripe motif that strongly influences the fabric’s anisotropic electromagnetic response. Figures 1(b) and 1(c) present photographs of the front (Side A) and back (Side B) surfaces of the carbon fabric, together with a schematic representation of the thread distribution. A schematic illustration of the final three-layer configuration is shown in Fig. 1(d), and the detailed compositions of the fabricated samples are summarized in Table 1.

**Fig. 2:**
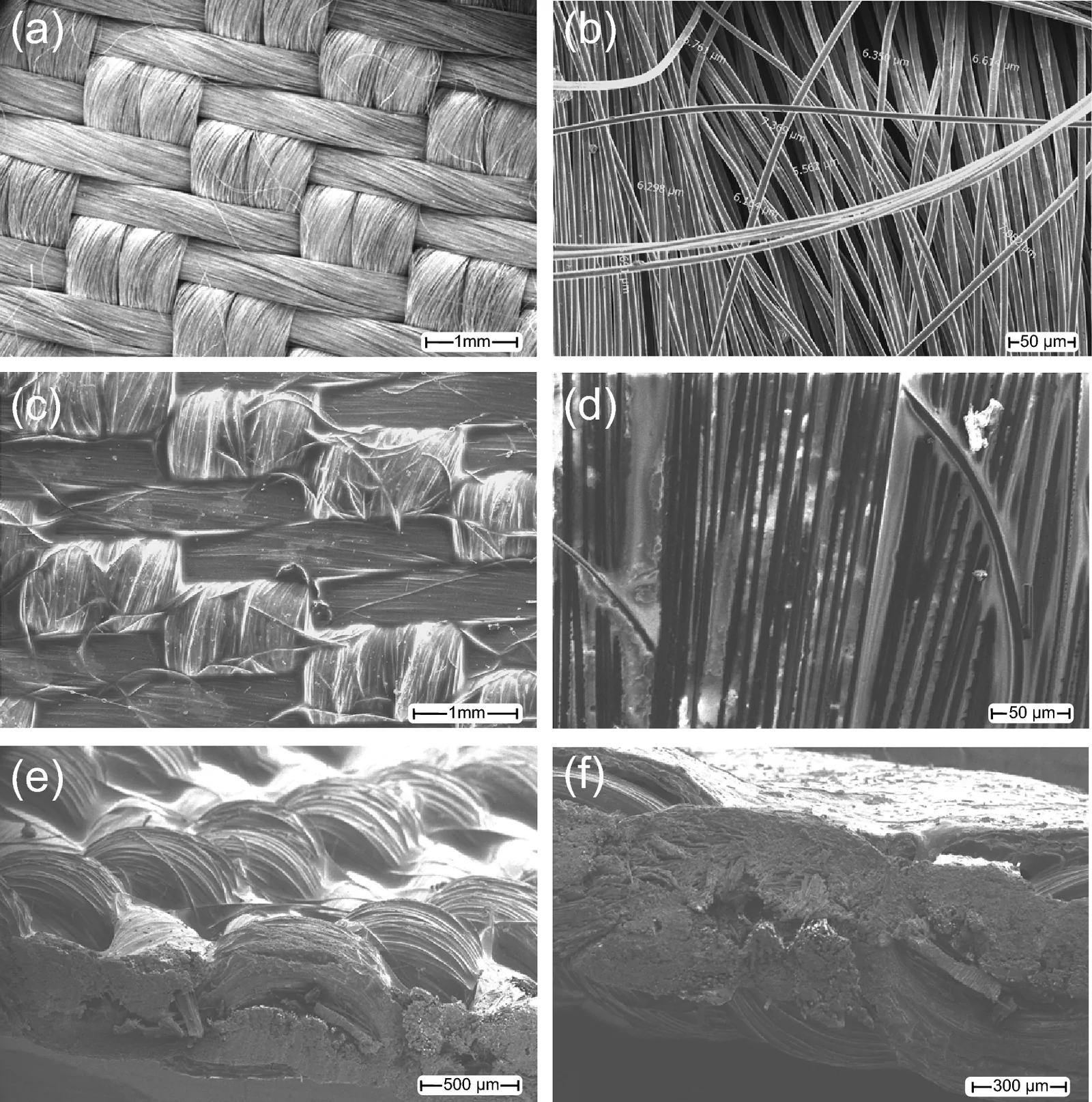
SEM images of the carbon fabric and resulting composite. (**a**) Surface morphology of Side A of the carbon fabric, displaying the woven arrangement of the thread bundles. (**b**) High-magnification view of the fabric, resolving individual carbon filaments and bundle thicknesses. (**c**) Side A and (**d**) corresponding thread bundles coated with the epoxy-based film. (**e**, **f**) Cross-sectional views of the composite, illustrating the uniform embedding of the fabric within the polymer matrix and the orientation of the thread bundles. Together, these micrographs highlight the millimeter-scale periodicity of the weave and the micrometer-scale morphology of the filaments, confirming successful and homogenous matrix infiltration.

**Fig. 3:**
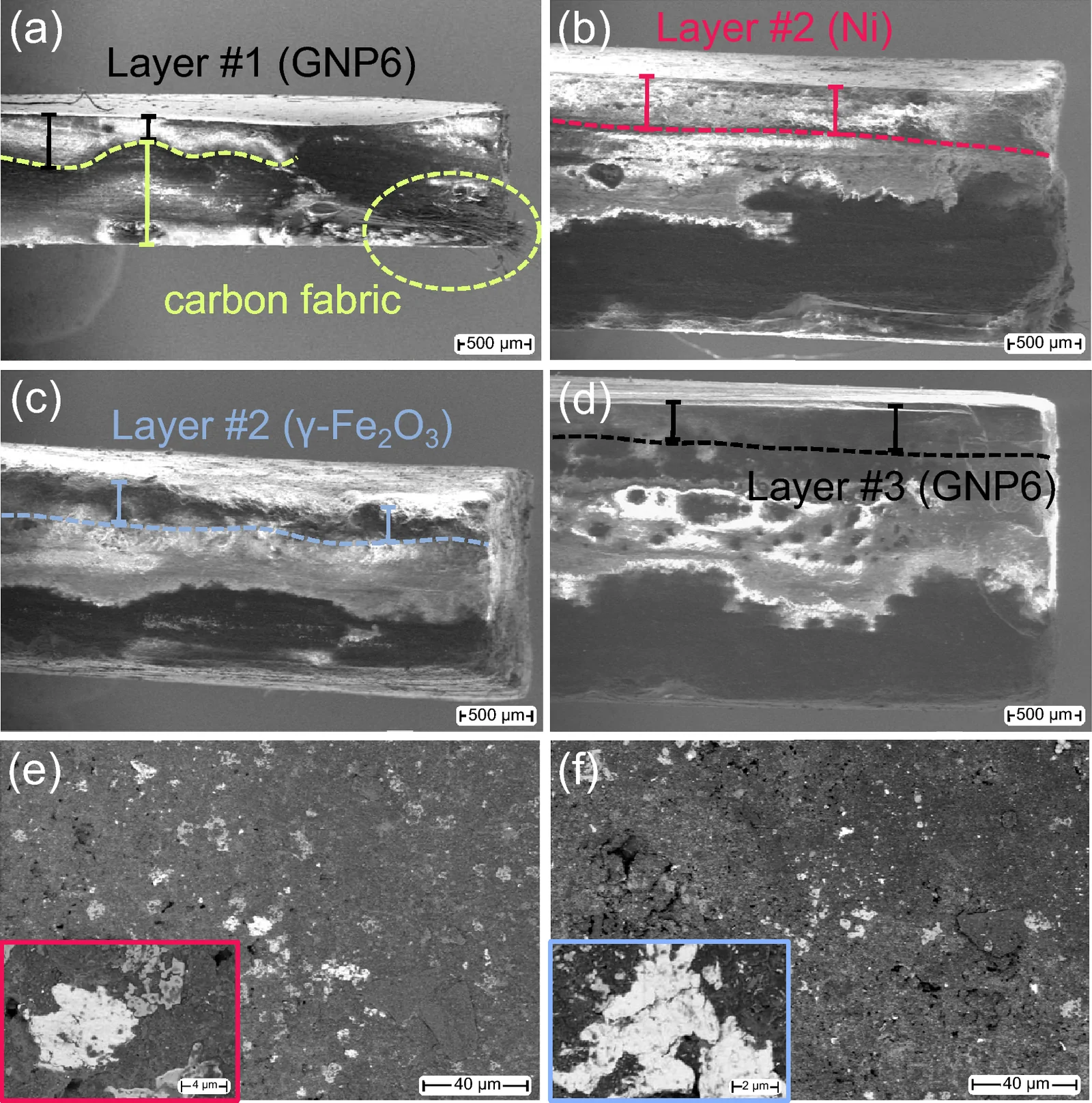
Cross-sectional SEM micrographs and structural details of the multilayered composite. Magnified views of (**a**) the arrangement of Layer #1 (GNP6) upon the underlying carbon fabric substrate, (**b**) interface between Ni-based Layer #2 and Layer #1, (**c**) interface between *γ*-Fe$_{2}$O$_{3}$-based Layer #2 and Layer #1, and (**d**) interface between GNP6 Layer #3 and Layer #2 (scale bars: 500 *μ*m). Ultra-high magnification micrographs showing the fine surface of (**e**) Ni and (**f**) *γ*-Fe$_{2}$O$_{3}$ layers (scale bars: 40 *μ*m).

Figures 2 and 3 provide SEM images of the carbon fabric and composite. It further illustrates both the millimeter-scale periodicity of the weave and the micrometer-scale morphology of the individual filaments, confirming uniform matrix infiltration and preservation of the fabric’s structural integrity within the composite.

Although SEM imaging primarily serves to verify the integrity and uniformity of the multilayer structure, the observed continuous, well-defined interfaces between the carbon fabric and successive polymer layers are also relevant to electromagnetic loss mechanisms. These interfaces act as sites for interfacial polarization and scattering, which may contribute to dielectric relaxation losses at millimeter-wave and sub-terahertz frequencies. Furthermore, the embedding of the woven fabric within the polymer matrix creates a tortuous internal geometry that facilitates multiple reflections and repeated interaction of the electromagnetic wave with lossy constituents.

### Setup and measurement technique

**Fig. 4:**
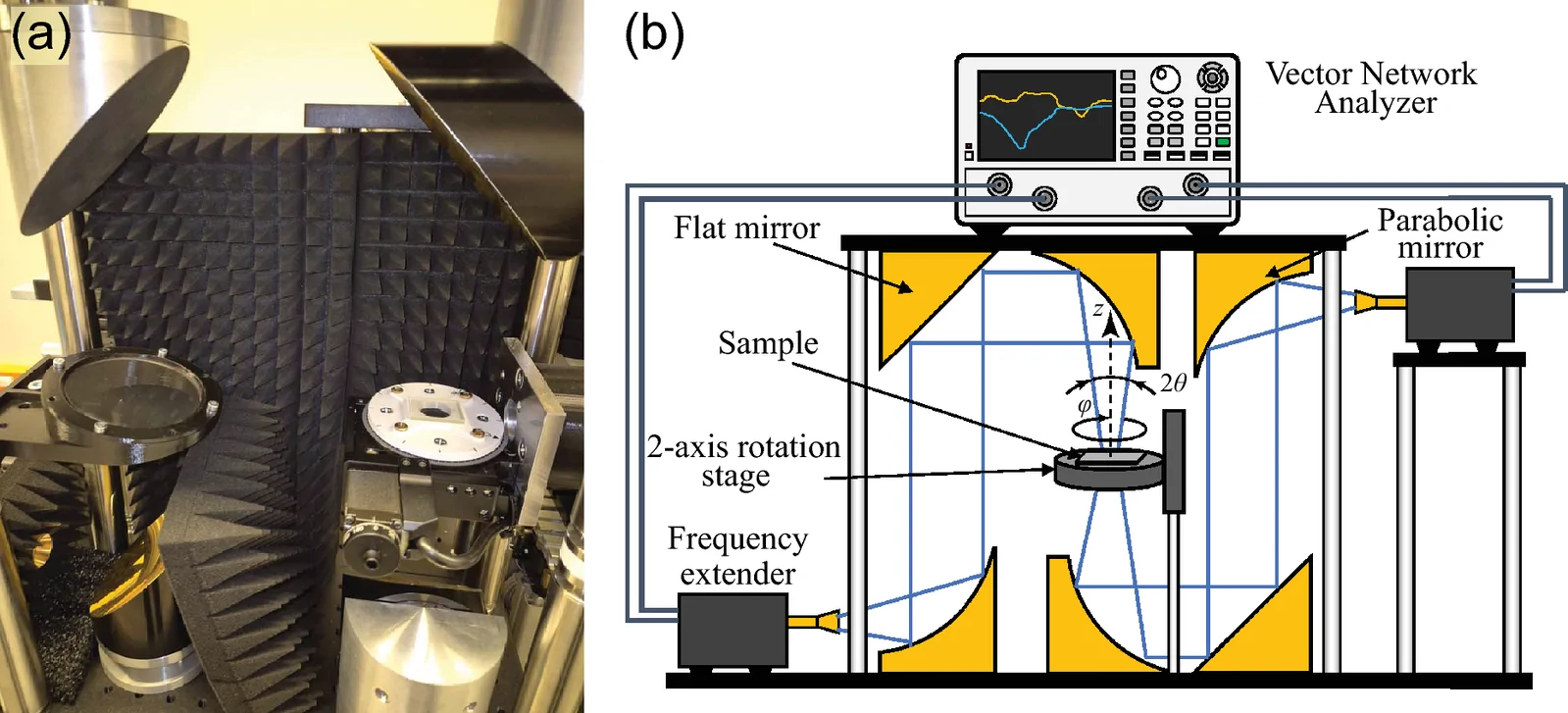
Free-space quasi-optical setup for shielding efficiency measurements. (**a**) Photograph of the experimental arrangement, showing horn antennas, parabolic mirrors, and the sample holder. (**b**) Corresponding schematic diagram of the optical path. The stage enables controlled rotation with respect to polarization angle *φ* and angle of incidence *θ*. Measurements were performed over the *90-330* GHz range using a VNA with frequency extenders. The beamwidth at the sample plane is approximately *15-20* mm, depending on frequency.

The multilayer configuration was intentionally constructed as a non-optimized but physically guided system to illustrate the interplay between conductive, magnetic, and geometric loss mechanisms. Rather than maximizing shielding effectiveness through extensive compositional tuning, the present design emphasizes clarity of physical interpretation, allowing the individual and combined contributions of the carbon fabric metastructure, conductive graphite layers, and magnetic particle-loaded layers to be systematically evaluated.

The shielding efficiency of the composites was evaluated based on the scattering parameters obtained using a free-space quasi-optical measurement setup, as shown in Fig. 4. The setup consists of a vector network analyzer Agilent PNA-X N5245A (VNA) equipped with frequency extenders from Virginia Diodes Inc., two conical horn antennas, and a set of flat and off-axis parabolic mirrors. The quasi-optical geometry provides a collimated beam path and minimizes spurious reflections, enabling accurate characterization in the millimeter- and sub-terahertz frequency ranges.

The parabolic mirrors focus the electromagnetic beam onto the sample under test. At the sample plane, the beamwidth measures approximately *15-20* mm, depending on the operating frequency. Each horn antenna is positioned so that its phase center coincides with the focal point of the corresponding parabolic mirror, ensuring optimal beam collimation. The focal points of the remaining mirrors converge at the sample holder, which is mounted on a two-axis motorized rotary stage.

The rotary stage provides precise control of the sample orientation. Rotation about the first axis (defined by the azimuth angle *φ*), which is perpendicular to the propagation direction of the incident wave, adjusts the alignment of the sample relative to the polarization of the incident field. Rotation about the second axis (defined by the elevation angle *θ*), changes the angle of incidence. In this study, only the first axis is utilized ($\varphi \in [0,360^\circ ]$, $\theta \approx 0^\circ$), as small variations in incidence angle are found to have negligible influence on shielding efficiency.

Measurements are performed over the frequency range *f ∈ [90, 330]* GHz, which covers the WR-8, WR-5, and WR-3 waveguide bands. The frequency resolution is set to 10 MHz, and the VNA configuration provides a dynamic range of approximately 100 dB. This wide dynamic range ensures accurate measurements even for composites with very high shielding efficiency. For each sample under study, complex scattering parameters ($S_{11}$, $S_{21}$) are recorded in angular increments of $\varphi = 5^\circ$.

To improve measurement accuracy, time-domain gating is applied to suppress unwanted reflections from elements of the quasi-optical path. In this procedure, the measured frequency-domain scattering parameters are first transformed into the time domain using an inverse Fourier transform^46^. An appropriate time window is then applied to isolate the response of the sample and remove delayed reflections from the setup. Finally, the data are transformed back into the frequency domain, yielding reflection and transmission coefficients that more accurately represent the intrinsic response of the material.

This combination of free-space quasi-optical techniques, wideband frequency coverage, and time-domain post-processing ensures that the measured data reliably capture the shielding performance of the composite absorbers across the millimeter-wave and sub-terahertz ranges.

Finally, to compensate for transmission losses and determine the reflection and transmission coefficients, two reference measurements are performed: one without the sample and another with a metallic plate (mirror). Using these references, the transmission (*T*), reflection (*R*), absorption (*A*), and effective absorption ($A_\text {eff}$) coefficients are calculated according to standard equations^47^:

1 $$\begin{aligned} \begin{aligned} T(\varphi ,f)&=\frac{|S_{21s}(\varphi ,f)|^2}{|S_{21e}(\varphi ,f)|^2}, \\ R(\varphi ,f)&=\frac{|S_{11s}(\varphi ,f)|^2}{|S_{11m}(\varphi ,f)|^2}, \\ A(\varphi ,f)&=1 - R(\varphi ,f) - T(\varphi ,f), \\ A_\text {eff}(\varphi ,f)&=\frac{A(\varphi ,f)}{1-R(\varphi ,f)}, \end{aligned} \end{aligned}$$

where $S_{11s}(\varphi ,f)$ and $S_{21s}(\varphi ,f)$ are complex scattering parameters for the measurements with the sample, $S_{21e}(\varphi ,f)$ is the result for the measurement without the sample in the optical path, and $S_{11m}(\varphi ,f)$ is the result for the measurement with a reference metallic mirror.

The shielding efficiency (SE) describes the performance of the shield, and it is defined as^48^:

2 $$\begin{aligned} \text {SE}=-10\log \frac{\text {incident power density}}{\text {transmitted power density}}, \end{aligned}$$

where the two power densities in this ratio correspond to the measured powers before and after placing the shield.

Coefficients (1) can then be used for the calculation of the shielding efficiency parameters using the following equations:

3 $$\begin{aligned} \begin{aligned} \text {SE}_R(\varphi ,f)&=-10\log [1-R(\varphi ,f)], \\ \text {SE}_A(\varphi ,f)&=-10\log [1-A_\text {eff}(\varphi ,f)], \\ \text {SE}_\text {total}(\varphi ,f)&=\text {SE}_R(\varphi ,f)+\text {SE}_A(\varphi ,f), \end{aligned} \end{aligned}$$

where $\text {SE}_R(\varphi ,f)$, $\text {SE}_A(\varphi ,f)$, and $\text {SE}_\text {total}(\varphi ,f)$ are the reflection, absorption, and total shielding efficiencies, respectively.

The measured transmission and reflection data were also used to retrieve effective electromagnetic parameters for use in theoretical modeling. For each composite layer, the complex transmission ($S_{21}$) and reflection ($S_{11}$) coefficients were obtained from free-space quasi-optical measurements.

## Results and discussion

### Experimental characterization

The principle of reciprocity in electromagnetic wave propagation states that, for a linear, time-invariant, and reciprocal medium, the transmission characteristics between two ports remain identical regardless of the direction of propagation^49^. Applied to multilayer structures, this implies that the transmission coefficient through the stack is the same whether the wave is incident from Side A or Side B.

When absorption is present in the layers, reciprocity still holds: the total transmitted power for a wave propagating forward is equal to that for a wave propagating in reverse. However, the reflection behavior may differ significantly depending on the direction of incidence, because the field distributions within the layers depend on the sequence of materials. As a result, while the transmission shielding efficiency $\text {SE}_T$ is invariant under wave reversal, the reflection-related efficiencies (e.g., $\text {SE}_R$ and $\text {SE}_A$) can differ for opposite irradiation directions.

This distinction is particularly important for asymmetric multilayer stacks, where the order of lossy and reflective layers leads to different reflection responses, but reciprocity guarantees that the absorption and transmission balance is preserved between the two directions.

The use of a full two-port measurement setup makes it possible to evaluate the shielding performance of the samples from both directions of incidence simultaneously. This capability is particularly important because the prepared specimens are structurally asymmetric, and therefore efficiencies $\text {SE}_R$ and $\text {SE}_A$ can depend on whether the wave impinges from Side A or from Side B. In contrast, the transmission shielding efficiency, $\text {SE}_T$, is constrained by the reciprocity principle and thus remains identical for forward and reverse propagation. For this reason, the discussion that follows concentrates on the reflection and absorption contributions only.

**Fig. 5:**
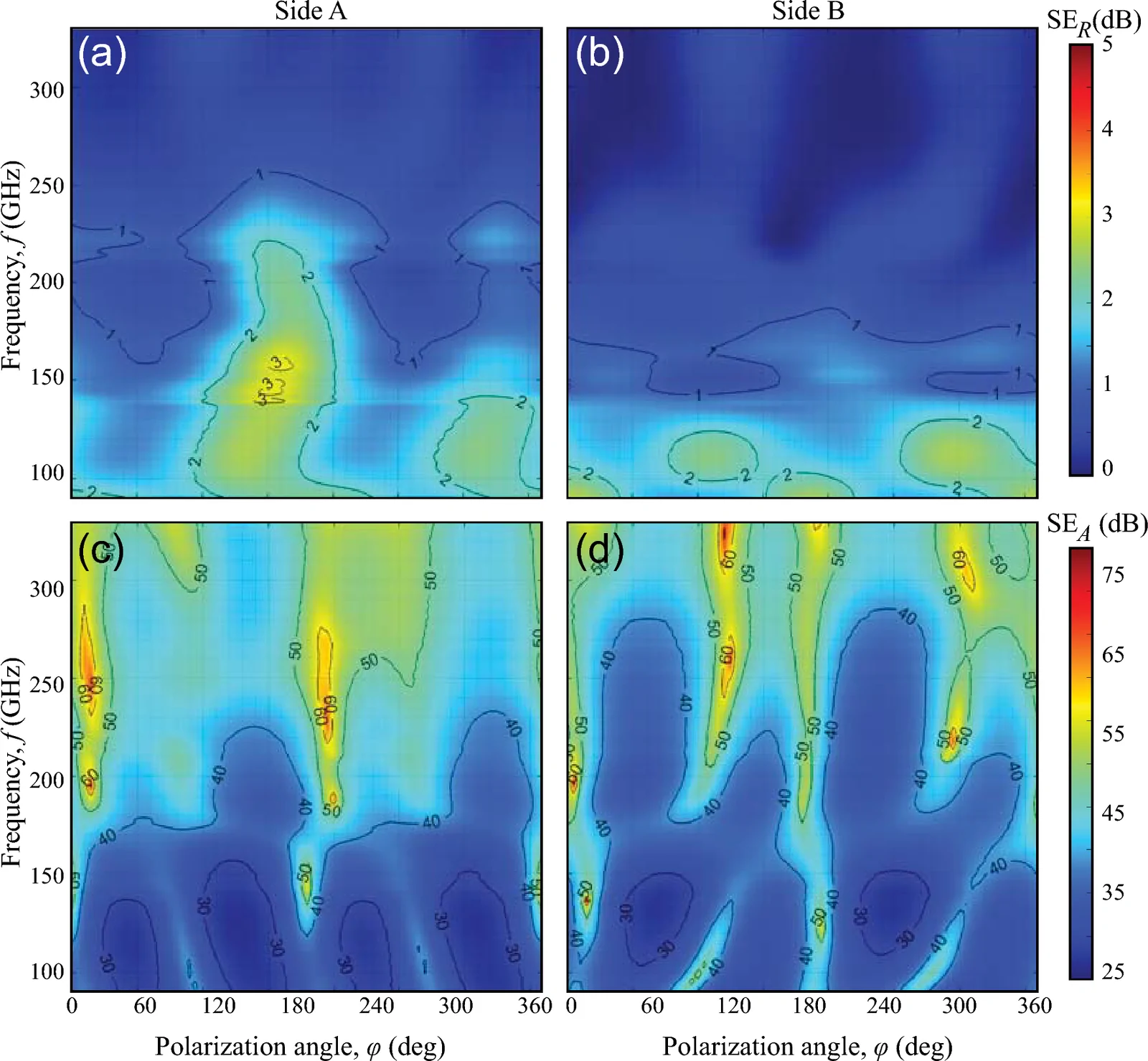
Reflection and absorption shielding efficiencies of a single-layer composite (Sample #1). Panels (**a**) and (**b**) show reflection efficiency $\text {SE}_R$ for irradiation from Side A and Side B, respectively; panels (**c**) and (**d**) show absorption efficiency $\text {SE}_A$ for the two sides. Efficiencies are plotted as functions of polarization angle *φ* ($0-360^\circ$, horizontal axis) and frequency *f* (*90-330* GHz, vertical axis). Color scales indicate efficiency in decibels (dB), with reflection typically in the range *0-5* dB and absorption extending up to *∼ 60* dB.

**Fig. 6:**
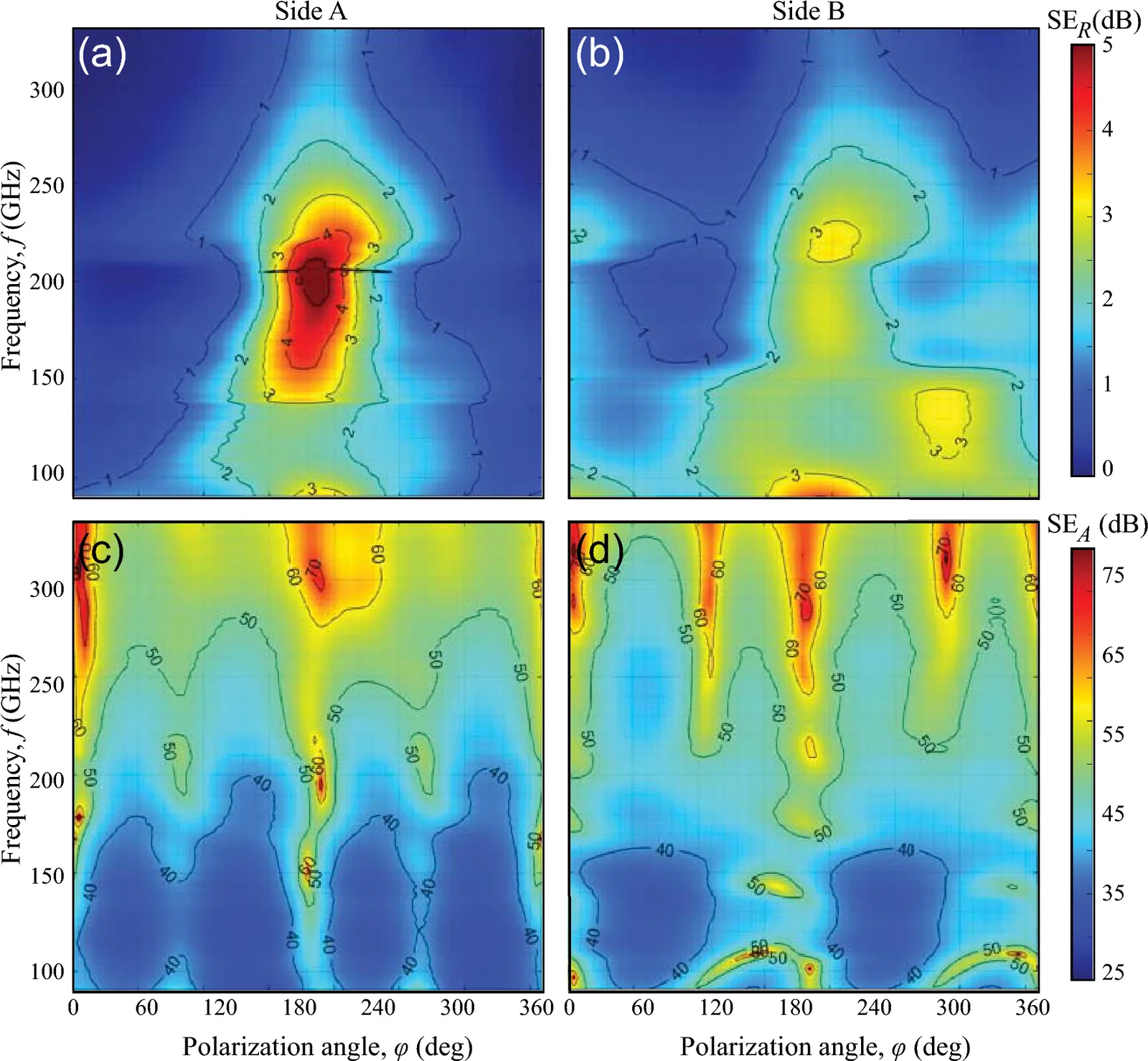
Reflection and absorption shielding efficiencies of a two-layer composite (Sample #2, with Ni filler in Layer #2). Panel organization is the same as in Fig. 5. Reflection remains generally low but exhibits localized peaks, while absorption broadens and strengthens compared to Sample #1. Color scales represent efficiency in decibels: reflection *∼ 0-10* dB, absorption up to *∼ 70* dB.

**Fig. 7:**
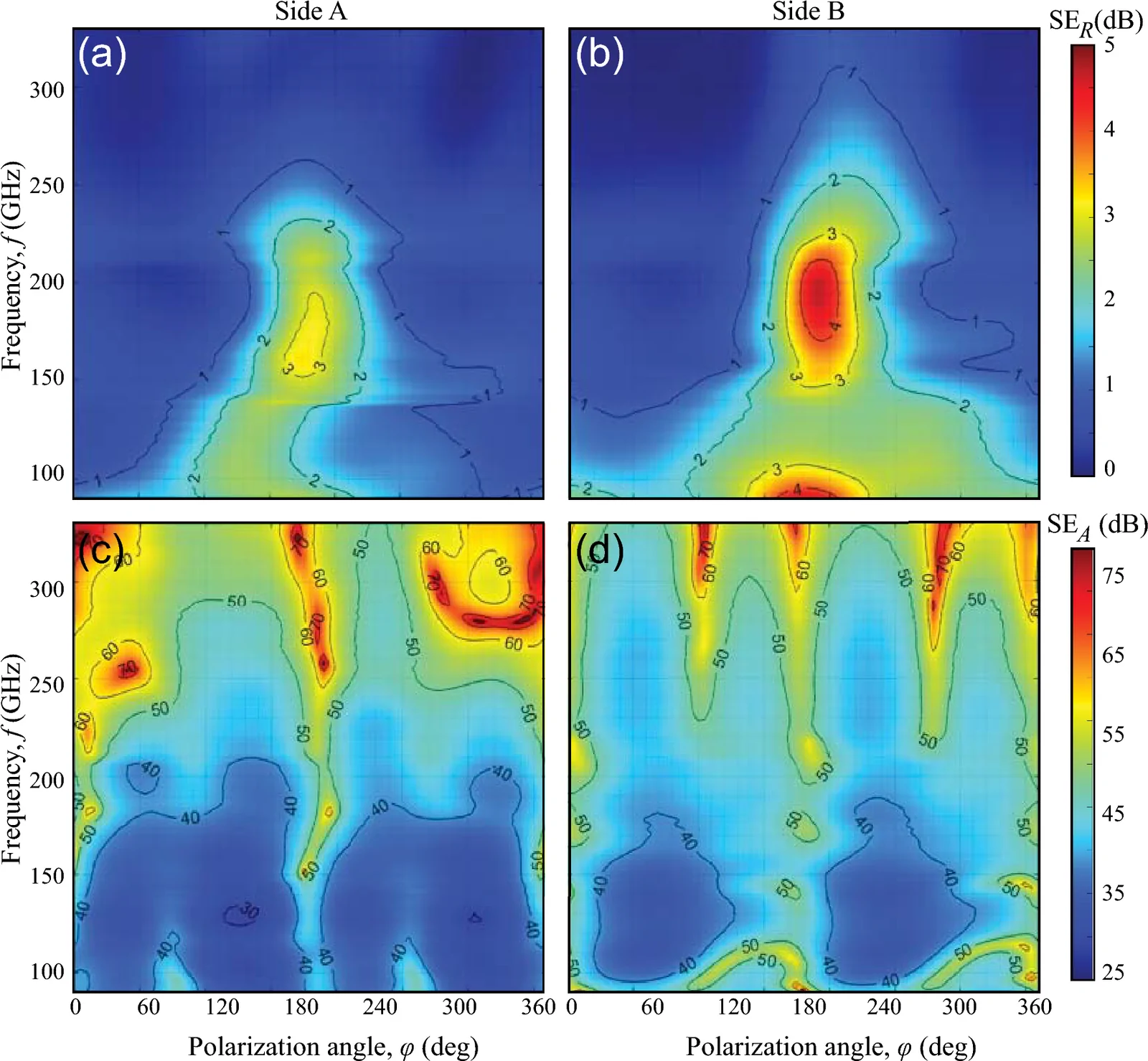
Reflection and absorption shielding efficiencies of a three-layer composite (Sample #3, with Fe$_2$O$_3$ filler in Layer #2 and an additional graphite outer layer). Panel organization is the same as in Figs. 5 and 6. Reflection is minimized across much of the *250-330* GHz range, while absorption reaches peak efficiencies of *∼ 75-80* dB. Color scales indicate efficiency in decibels, with reflection typically *<5* dB and absorption spanning a wide and high-efficiency range.

The results of our measurements are summarized in Figs. 5,6,7, which present the efficiencies for the three representative samples. In each figure, the efficiencies are plotted as functions in the $\{\varphi , f\}$ parameter space. The left panels correspond to irradiation from Side A, whereas the right panels show the response for incidence from Side B, highlighting how structural asymmetry influences the balance between reflection and absorption.

The observed angular dependences of the shielding efficiency originate from the intrinsic anisotropy of the fabric metastructure (one vs two threads per period). The diagonal orientation of the bundles leads to two-fold rotational symmetry in the electromagnetic response, with polarization-dependent resonances aligned along and across the twill direction, such that the measured efficiency is invariant under a $180^\circ$ rotation of the polarization angle *φ*. Specifically, when the incident wave is linearly polarized along the *x*-axis ($\varphi = 0^\circ$), the electric field is aligned with the direction containing a single thread per period. In contrast, at $\varphi = 180^\circ$, the wave is polarized along the *y*-axis, where the fabric density is effectively doubled due to the presence of two threads per period. This difference in thread density along orthogonal directions gives rise to distinct electromagnetic responses for the two principal polarizations. For intermediate values of *φ*, the incident polarization is oriented diagonally with respect to the weave, thereby exciting both thread orientations simultaneously. As a result, the efficiency maps display a continuous angular modulation between these two limiting cases, reflecting the anisotropic effective permittivity and impedance of the fabric. Nevertheless, in the experimental data, this symmetry is somewhat violated due to the irregularity of the weaving and orientation of the sample in accordance with the incident wave polarization and the diagonal pattern of bundles.

For Sample #1 (single coating layer; Fig. 5), reflection is uniformly very low across most of the band (colorbar scale *∼ 0-5* dB), with slight angle- and frequency-dependent ridges, where small reflection peaks align with certain *φ* and *f*. Reflection differs slightly between sides A and B, consistent with the asymmetric stack. The absorption level is already high (tens of dB) across wide portions of the band, with pronounced resonance bands whose position shifts with *φ*, consistent with structural and dielectric resonances of the fabric plus coating.

For Sample #2 (two layers; Layer #2 containing Ni filler; Fig. 6), reflection remains low overall but shows more pronounced angle- and frequency-localized features, likely due to the increased conductivity and impedance mismatch introduced by Ni. The side-to-side differences are clearer, and the absorption bands broaden with higher overall efficiency, indicating magnetic losses from the Ni layer and improved impedance matching in the multilayer configuration.

For Sample #3 (three layers; Layer #2 with Fe$_2$O$_3$ and an additional outer graphite layer; Fig. 7), reflection is minimal across much of the upper-frequency range (*>250* GHz), with only narrow reflection ridges at specific values of *f* and *φ*. The absorption maps reveal the strongest and broadest attenuation, with peaks reaching *∼ 75-80* dB and resonance bands merging into continuous regions of high absorption across wide *φ* intervals. Side A vs Side B differences remain, as expected for an asymmetric multilayer structure.

The progressive increase from single-layer to multilayer architectures has a direct impact on the dominant electromagnetic loss mechanisms. In the single-layer configuration (Sample #1), attenuation is governed primarily by dielectric relaxation and ohmic losses associated with the conductive carbon fabric and the graphite-filled polymer coating. The limited number of internal interfaces restricts interfacial polarization and internal reflections, resulting in discrete, polarization-dependent absorption bands.

In the two-layer structure (Sample #2), the introduction of a magnetic particle-loaded polymer layer adds a loss channel through magnetic permeability dispersion, while simultaneously increasing the number of internal interfaces. These interfaces enhance dielectric relaxation losses. Moreover, the impedance contrast between layers increases internal reflections, extending the effective propagation path of the electromagnetic wave and broadening the absorption bands.

The three-layer configuration (Sample #3) further amplifies these effects. The additional outer graphite-filled layer creates a more gradual impedance transition from free space to the conductive fabric substrate, improving impedance matching and reducing front-surface reflection. At the same time, the increased number of interfaces and layer thickness promotes multiple internal reflections and interference effects, effectively trapping electromagnetic waves within the structure and enhancing cumulative dielectric and magnetic losses. As a result, individual resonances merge into extended regions of high absorption across wide frequency and polarization ranges.

**Fig. 8:**
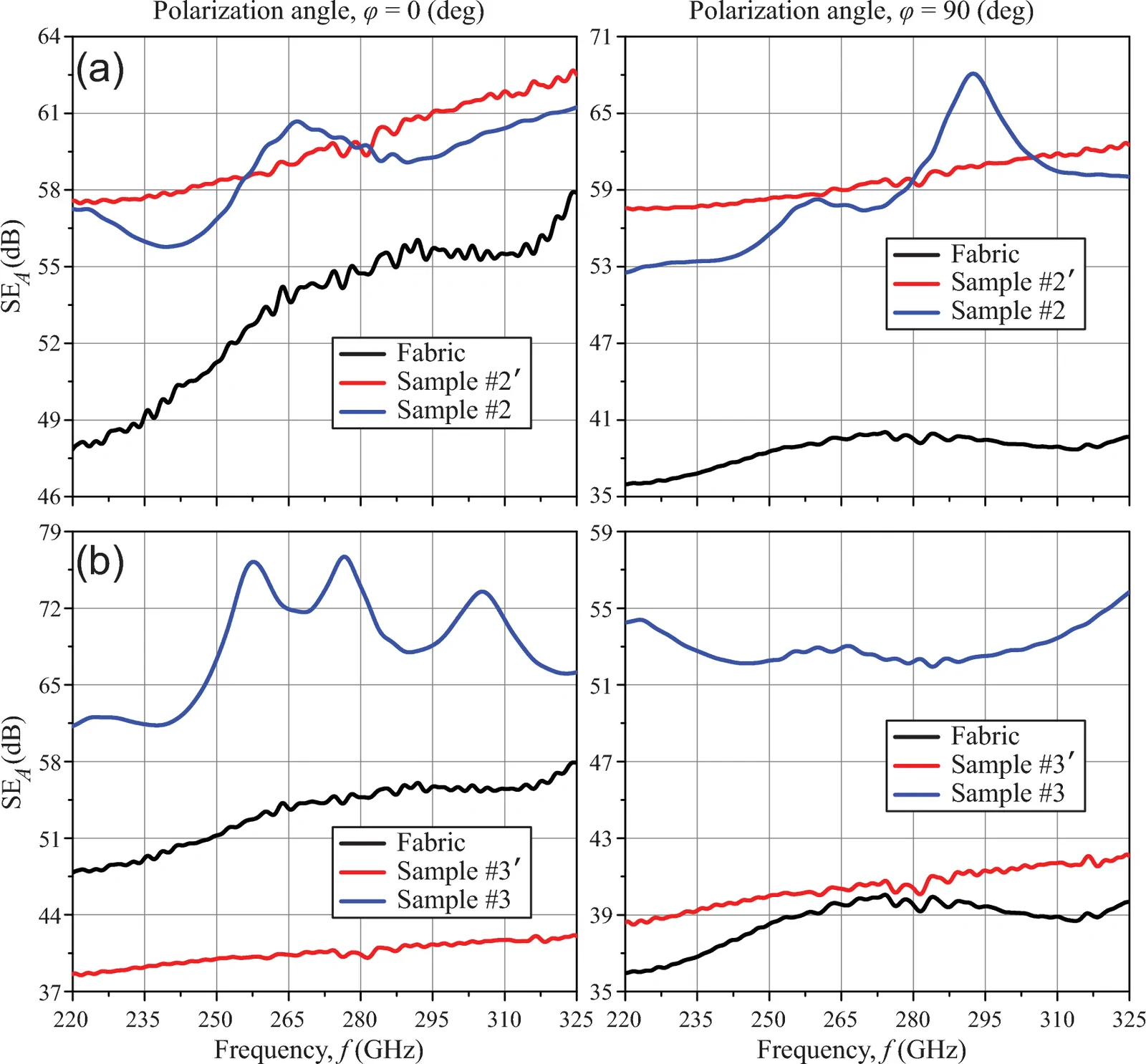
Dependence of the absorption shielding effectiveness ($\text {SE}_A$) on frequency in the range of *220-325* GHz for polarization angles of $\varphi = 0^\circ$ (left column) and $\varphi = 90^\circ$ (right column). Comparisons are shown for (**a**) Sample #2 and auxiliary Sample #2$^\prime$, and (**b**) Sample #3 and auxiliary Sample #3$^\prime$ (see, Tab. 1). The black curves represent the base fabric material.

The observed evolution from discrete absorption bands in single-layer samples to broadband absorption in multilayer samples directly reflects the compositional complexity of the structure. As additional conductive and magnetic layers are introduced, overlapping dielectric and magnetic loss mechanisms lead to the merging of individual resonances into extended high-absorption regions. This behavior indicates that absorption enhancement arises not only from increased thickness, but from the spatial distribution and coupling of different loss mechanisms enabled by the multilayer composition (measured characteristics of the intermediate structures can be found in Supplementary Information, see Appendix A).

A precise quantitative evaluation of this spectral behavior is illustrated in Fig. 8, which contrasts the absorption shielding effectiveness ($\text {SE}_A$) profiles of the main composites against their auxiliary iterations at distinct polarization alignments ($\varphi = 0^\circ$ and $\varphi = 90^\circ$). As seen in Fig. 8(a), the complete double-layer structure (Sample #2) demonstrates a clear enhancement over the auxiliary Sample #2$^\prime$ across the *220-325* GHz band. This trend is even more pronounced in the three-layer system presented in Fig. 8(b), where Sample #3 significantly outperforms the auxiliary Sample #3$^\prime$, revealing that the inclusion of Layer #1 is vital for establishing optimal impedance matching and resonant absorption.

It should be noted that the increase in total shielding effectiveness from Sample #1 to Sample #3 cannot be attributed solely to increased thickness. Although the total thickness increases from 1.3 to 1.8 mm, the experimental results also reveal substantial changes in spectral shape, reflection suppression, and polarization-dependent resonance behavior, indicating that the redistribution of conductive and magnetic loss channels within the multilayer architecture plays a dominant role.

Taken together, these results demonstrate that all three composites are strongly absorption-dominant ($\text {SE}_A \gg \text {SE}_R$) across the entire *90-330* GHz band. Moreover, the systematic addition of magnetic and conductive filler layers (Ni, Fe$_2$O$_3$, graphite) progressively enhances both the strength and bandwidth of absorption, while simultaneously suppressing reflection. The observed angular anisotropy, governed by the fabric weave, further illustrates how structural design can be harnessed to tailor the electromagnetic response. These findings confirm that multilayer carbon-based composites can achieve broadband, polarization-sensitive, and absorption-dominated shielding, making them promising candidates for advanced high-frequency electromagnetic protection applications.

It is important to distinguish the observed enhancement from a purely cumulative attenuation effect associated with increasing the number of lossy layers. If the performance improvement were solely additive, one would expect a relatively uniform increase in shielding effectiveness with thickness, without significant modification of the spectral or angular features. In contrast, the introduction of the magnetic layer leads to the emergence and broadening of absorption bands, a pronounced suppression of reflection, and a reshaping of polarization-dependent resonances governed by the fabric metastructure. These features indicate that magnetic losses are not acting independently, but are coupled to the geometric and conductive resonances of the carbon fabric, producing a non-additive response.

### Effect of multilayer composition

The absorption performance of the composite is strongly influenced by the composition and sequence of the multilayer structure. In the present design, the graphite-filled polymer layers provide moderate electrical conductivity and broadband dielectric loss, which promote ohmic dissipation while maintaining impedance matching at the air-material interface. Excessively high conductivity would lead to dominant reflection; therefore, the selected graphite concentration balances dielectric loss and wave penetration.

The electronic transport and dielectric response of the intermediate layers are phenomenologically integrated into the macroscopic scattering behavior of the composite. The 6 wt.% graphite loading is chosen to target a moderate conductivity regime, enabling wave penetration and optimizing the balance between surface reflection and internal ohmic dissipation.

The incorporation of a magnetic particle-loaded polymer layer introduces an additional absorption mechanism through magnetic permeability dispersion and associated magnetic loss. Although the intrinsic magnetic resonances of Ni and Fe$_2$O$_3$ occur at lower frequencies, their frequency-dependent permeability contributes to loss broadening and impedance matching in the millimeter-wave and sub-terahertz ranges when combined with dielectric losses.

Importantly, the sequence of conductive and magnetic layers creates a graded electromagnetic response across the thickness of the composite. Incident waves experience a gradual transition from free space into regions of increasing dielectric and magnetic loss, reducing front-surface reflection and enabling deeper penetration into the structure. Once inside, electromagnetic waves undergo multiple internal reflections and repeated interactions with lossy constituents, leading to enhanced absorption. This compositionally graded loss distribution explains the superior absorption performance of the multilayer structure compared to single-composition or single-layer samples.

### Retrieval of effective electromagnetic parameters

To further interpret the experimentally observed shielding behavior, the measured complex transmission ($S_{21}$) and reflection ($S_{11}$) coefficients were used to estimate the effective electromagnetic parameters of the multilayer composites. Because the investigated structures consist of multiple conductive and magnetic layers with finite thicknesses and internal interfaces, the retrieval was performed within an effective-medium approximation using a standard inversion procedure adapted for free-space measurements of stratified samples^50^. The resulting effective complex permittivity $\varepsilon _\textrm{eff}(\omega )$ and permeability $\mu _\textrm{eff}(\omega )$ therefore represent equivalent constitutive parameters of the entire multilayer structure rather than intrinsic parameters of an individual homogeneous layer.

The effective constitutive parameters were obtained from the retrieved effective refractive index $n_\textrm{eff}$ and normalized impedance $z_\textrm{eff}$ according to

4 $$\begin{aligned} \varepsilon _\textrm{eff}(\omega )=\frac{n_\textrm{eff}(\omega )}{z_\textrm{eff}(\omega )}, \qquad \mu _\textrm{eff}(\omega )=n_\textrm{eff}(\omega )z_\textrm{eff}(\omega ). \end{aligned}$$

The quantities $n_\textrm{eff}$ and $z_\textrm{eff}$were estimated numerically from the measured scattering parameters using the inversion procedure described in Ref^50^., adapted for finite-thickness stratified structures measured in a free-space quasi-optical configuration.

The retrieved parameters reveal that the electromagnetic response of the composites is dominated by strong dielectric losses associated with the conductive carbon fabric and graphite-filled polymer layers. In particular, the imaginary component of $\varepsilon _\textrm{eff}$ increases in the multilayer configurations, indicating enhanced conductive and interfacial polarization losses. In samples containing Ni and *γ*-Fe$_2$O$_3$ fillers, a frequency-dependent permeability dispersion is additionally observed. The magnetic layers also increase the imaginary component of $\mu _\textrm{eff}$, indicating additional magnetic dissipation channels that contribute to absorption enhancement, impedance matching, and absorption broadening in the millimeter-wave and sub-terahertz frequency range.

The retrieved effective parameters were used as physically motivated input estimates for the transfer-matrix calculations discussed below. Importantly, the same parameter sets were consistently applied across all simulations without independent fitting for individual samples, ensuring that the calculated differences arise from changes in multilayer architecture rather than arbitrary parameter adjustment.

It should be noted that, for structurally asymmetric multilayer composites with coupled dielectric, magnetic, and geometric resonances, the retrieval of unique constitutive parameters from free-space scattering data represents a nontrivial inverse problem. In the present work, the extracted parameters are therefore interpreted within an effective-medium approximation. A rigorous inverse reconstruction accounting explicitly for internal interfaces, anisotropy, and polarization-dependent coupling effects is beyond the scope of the present study and will be addressed in future work.

### Theoretical description

To interpret and capture the essential mechanisms governing the high-frequency response of the proposed composites, a theoretical model was developed based on the transfer-matrix formalism. The model is constructed in a modular framework to simultaneously account for: (i) the anisotropic metastructure of the woven carbon fabric, (ii) the multilayer coating architecture, (iii) effective material dispersion including sub-THz magnetic dissipation paths, and (iv) multiple reflections and geometric interference effects within the stack.

The transfer-matrix method represents the full composite as an assembly of *N=4* planar layers, where the woven fabric is treated as a structurally anisotropic effective medium and the coating layers are modeled as homogeneous slabs. By construction, this 1D formulation preserves reciprocity. For plane waves at near-normal incidence, the characteristic propagation matrix $\textbf{M}_j$ of the *j*-th layer (with thickness $a_j$, complex refractive index $n_j=\sqrt{\varepsilon _j\mu _j}$, and wave impedance $Z_j=\sqrt{\mu _j/\varepsilon _j}$) is given by:

5 $$\begin{aligned} \textbf{M}_j = \begin{pmatrix} \cos \beta _j & i Z_j \sin \beta _j\\ i Z_j^{-1}\sin \beta _j & \cos \beta _j \end{pmatrix}, \end{aligned}$$

where $\beta _j = k_0 n_j a_j$, $k_0=\omega /c$, and $\omega =2\pi f$. The total transfer matrix of the multilayer stack is obtained via sequential multiplication, $\textbf{M}=\prod _j \textbf{M}_j$. From the global matrix $\textbf{M}$, the complex reflection (*ρ*) and transmission (*τ*) coefficients are calculated relative to the free-space impedance $Z_0$, yielding the power coefficients $R=|\rho |^2$ and $T=|\tau |^2$. Using these values, the shielding efficiencies $\textrm{SE}_R$, $\textrm{SE}_A$, and $\textrm{SE}_T$ are derived consistently with the experimental definitions established in Eq. (1).

The structural anisotropy introduced by the woven layout of the carbon fabric is modeled via an angle-dependent effective permittivity and surface impedance. To first order, this is represented by a rotated in-plane uniaxial tensor:

6 $$\begin{aligned} \varepsilon _{\text {eff}}(\varphi ,f) \approx \varepsilon _{\perp }(f) + \Delta \varepsilon (f)\cos (2\varphi ), \end{aligned}$$

with an analogous form assigned to $\mu _{\text {eff}}$. The $\cos (2\varphi )$ modulation reflects the two-fold rotational symmetry of the weave under linear polarization, establishing the angular dependency of $n_j(\varphi )$ and $Z_j(\varphi )$.

Conduction and dielectric relaxation losses within the fabric and graphite coatings are incorporated via the complex permittivity function:

7 $$\begin{aligned} \varepsilon _j(\omega )=\varepsilon _{j,\infty } + \sum _\ell \frac{\Delta \varepsilon _{j,\ell }}{1+i\gamma _{j,\ell }\omega } + i\frac{\sigma _j}{\varepsilon _0\omega }, \end{aligned}$$

where the final term accounts for ohmic dissipation. For the carbon fabric, representative low-frequency microwave benchmarks suggest a high-frequency permittivity background of $\varepsilon _{\infty }\approx 2.5-3.5$, relaxation strengths of $\Delta \varepsilon \approx 0.2-0.8$, and relaxation times of $\gamma \approx 10^{-9}-10^{-6}$ s, corresponding to interfacial and dipolar polarization at fiber junctions^51^. The effective electrical conductivity of carbon fabrics typically spans $\sigma \approx 10^{1}-10^{3}$ S/m, depending on fiber density and resin content^52^. For graphite coatings, larger values are typically expected: $\varepsilon _{\infty } \approx 3-5$, relaxation contributions of $\Delta \varepsilon \approx 0.1-0.3$ with $\gamma \approx 10^{-11}-10^{-9}$ s, and conductivities up to $\sigma \approx 10^{4}$ S/m, as observed in carbon-loaded matrices over the *1-14* GHz band^53^.

Because our operational domain extends into the sub-terahertz regime (*90-330* GHz), standard low-frequency metallic dispersion models must be adjusted to match the microscale realities of the composite^54^. Regarding the graphite nanoplatelet (GNP6) coatings, the complex conductivity in the sub-terahertz regime is known to deviate from ideal bulk metallic Drude behaviors due to the disordered network morphology and tunneling-dominated transport between flakes^55^. Because hopping conduction often manifests as a stable or slightly ascending AC conductivity trend up to sub-THz frequencies before relaxation dominates, the value derived from the microwave spectrum provides a highly reliable and mathematically consistent effective parameter for our transfer-matrix approximation.

Magnetic losses within the Ni- or *γ*-Fe$_2$O$_3$-bearing layers are initially parameterized using a Lorentz-type permeability profile:

8 $$\begin{aligned} \mu _j(\omega ) = 1 + \frac{\Delta \mu _j\,\omega _{0,j}^2}{\omega _{0,j}^2 - \omega ^2 - i\gamma _j\omega }, \end{aligned}$$

where $\omega _{0,j}$ represents the magnetic resonance frequency, $\gamma _j$ denotes the damping constant, and $\Delta \mu _j$ is the resonance strength. Typical values for standalone Ni spinels restrict the natural ferromagnetic resonance (NFMR) frequency $\omega _{0}/2\pi$ to the *1-10* GHz range, with $\Delta \mu \approx 0.5-2.5$ and damping $\gamma /2\pi \approx 0.1-2$ GHz^56^. For Fe$_2$O$_3$ phases, these peaks occur between *5-20* GHz, with $\Delta \mu \approx 0.2-1.0$ and $\gamma /2\pi \approx 0.5-5$ GHz^57,58^.

It is important to clarify that while the nominal resonance frequencies $\omega _0/2\pi$ of bulk ferromagnetic components typically peak in the lower microwave bands, the parameters utilized in Eq. (8) serve as a phenomenological effective-medium model. In the sub-THz regime (*90-330* GHz), the primary physical contributions to magnetic attenuation are no longer governed by standard domain-wall dynamics or natural ferromagnetic resonance. Instead, they are governed by the high-frequency tails of exchange-dominated spin-wave excitations, spin-lattice relaxation paths, and microstructural eddy currents induced within individual filler grains^59^. Consequently, the extracted $\mu _{\text {eff}}$ represents the integrated response of these high-frequency dissipation processes. While this effective parameterization accurately captures the collective macroscopic attenuation, separating these individual microscopic contributions remains an objective for future refined modeling frameworks.

Finally, geometric resonances associated with the periodicity and anisotropy of thread bundles are incorporated through an additional resonant contribution to $\varepsilon _{\text {eff}}$:

9 $$\begin{aligned} \Delta \varepsilon _{\text {geom}}(\omega ,\varphi ) \propto \frac{\Phi (\varphi )}{\omega _{g}^2 - \omega ^2 - i\Gamma _g\omega }, \end{aligned}$$

where $\omega _g \propto c/(n L)$ is the characteristic resonance frequency determined by the in-plane structural feature size *L*, and $\Phi (\varphi )$ encodes the polarization-dependent coupling to the fabric geometry.

**Fig. 9:**
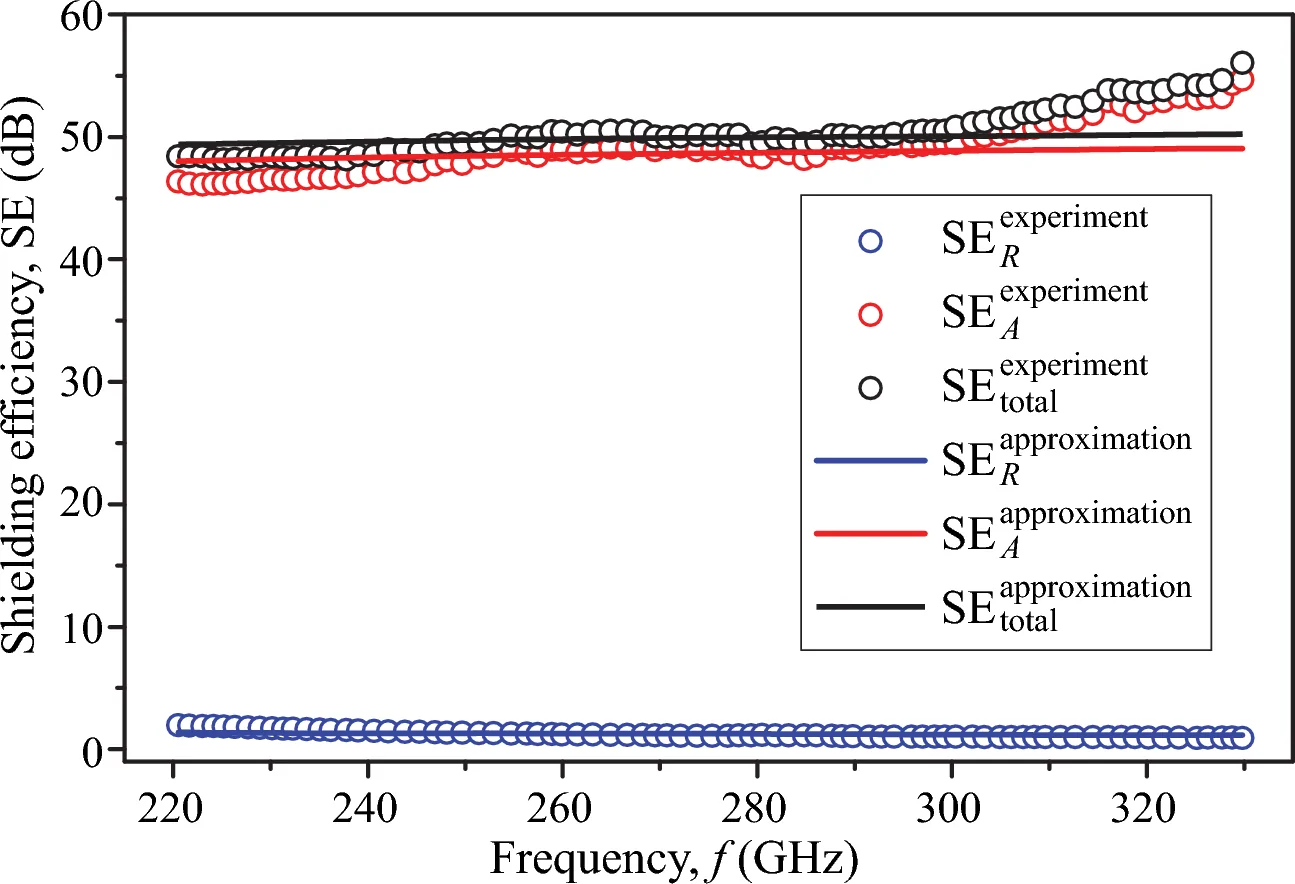
Comparison between experimental and theoretical results for the total ($\textrm{SE}_{\textrm{total}}$), absorption ($\textrm{SE}_A$), and reflection ($\textrm{SE}_R$) shielding efficiencies of the single-layer composite (Sample #1). Theoretical curves were calculated using the transfer-matrix model and effective constitutive relations [Eqs. (5)–(9)]. The strong correlation between measured and predicted data confirms that the model successfully reproduces the absorption-dominant behavior and frequency-dependent shielding performance of the composite in the *220-320* GHz range. For the carbon fabric, the following parameters were used: $\varepsilon _{\infty } = 3.5$, $\Delta \varepsilon = 0.207$, $\gamma = 1.150\times 10^{-7}$ s, and *σ = 24.4* S/m.

This theoretical framework successfully reproduces the prominent features observed in the experimental efficiency maps (Figs. 5–7), including the low reflection levels of the single-layer composite, the broadened absorption bands introduced by the Ni filler in the two-layer configuration, and the continuous, high-attenuation windows resulting from the combined *γ*-Fe$_2$O$_3$ and graphite layers in the three-layer sample. From a modeling perspective, transitioning from single- to multilayer configurations increases the number of impedance discontinuities and phase accumulation paths within the transfer-matrix framework. This structural evolution directly corresponds to the enhanced multiple-reflection losses and resonance broadening observed experimentally. By simultaneously accounting for anisotropy, high-frequency magnetic dissipation, and multilayer interference within a unified formulation, the model explains the side-dependent reflection asymmetry and functions as a predictive tool for designing absorption-dominant shields.

Figure 9 presents the calculated absorption performance of the single-layer composite as a reference case. Crucially, the retrieved electromagnetic parameters were not adjusted independently for each sample to artificially optimize agreement with the experiment. A single set of permittivity and permeability functions was assigned to each material layer type and applied consistently across all configuration permutations. This rigid approach ensures that variations in calculated performance arise strictly from changes in layer sequence and architecture, verifying the physical validity of the model. Note that for this single-layer configuration, the absorption can also be estimated using a simplified impedance-matching model. However, the full transfer-matrix calculation is presented here to validate the effective electromagnetic parameters and boundary conditions. This baseline validation ensures consistency before applying the framework to complex multilayer architectures, where simple impedance-matching approximations fail.

The strong agreement between the experimental results and the transfer-matrix predictions indicates that the framework accurately captures the dominant electromagnetic mechanisms responsible for wave attenuation. Both datasets reveal a commanding absorption component ($\textrm{SE}_A$) and minimal reflection ($\textrm{SE}_R$), confirming absorption-dominated performance across the *220-320* GHz range. Minor discrepancies appearing at the higher frequency boundary suggest that further model refinement could incorporate higher-order spatial harmonics and local microstructural scattering effects.

Furthermore, numerical simulations demonstrate that the experimentally observed absorption enhancement cannot be reproduced by treating the layers as isolated components with cumulative, additive losses. Instead, accurate replication requires accounting for the electromagnetic coupling between the magnetic permeability dispersion and the fabric-induced anisotropic permittivity, which cooperatively alter the internal resonance conditions and impedance matching across the stack. This collective behavior provides a clear theoretical basis for classifying the observed shielding enhancement as synergistic rather than purely additive.

### Benchmark comparison

To assess the advancement level of the proposed carbon fabric-magnetic multilayer composite, its EMR shielding performance is benchmarked against representative materials reported in the literature. The comparison emphasizes key figures of merit relevant for state-of-the-art shielding and absorber systems, including operating frequency range, maximum shielding effectiveness (SE), thickness, dominant attenuation mechanism, structural design, and practical limitations.

Table 2 summarizes the performance of conventional polymer-carbon composites, advanced carbon-based porous absorbers, magnetic filler-based materials, MXene-based shielding systems, metamaterial-inspired absorbers, and recently reported sub-terahertz electromagnetic shielding structures, alongside the present work. Most conventional EMI shielding materials operate primarily in the MHz–GHz range and rely predominantly on reflection as the main attenuation mechanism. Although carbon-based porous materials and magnetic composites improve absorption efficiency, they typically require relatively large thicknesses and exhibit limited effectiveness at millimeter-wave and sub-terahertz frequencies. Recently developed MXene-based structures demonstrate excellent conductivity and high shielding effectiveness, including ultrathin sub-terahertz shielding films and plasmonic metamaterial absorbers. However, their performance is often dominated by reflection losses, while long-term environmental stability and scalable fabrication remain challenging. In contrast, metamaterial-inspired and multilayer architectures provide improved impedance matching and enhanced absorption-dominated shielding through engineered resonant and interference effects.

In contrast, the composite presented in this work demonstrates exceptionally high, absorption-dominated shielding effectiveness of up to *∼ 80* dB in the *90-330* GHz range at a total thickness of only *∼ 1-2* mm. This performance surpasses that of conventional polymer-, carbon-, and magnetic-based shielding systems and rivals or exceeds that of far more complex metamaterial-based absorbers, while maintaining mechanical flexibility and scalable fabrication.

The benchmark comparison confirms that the synergistic integration of a woven carbon fabric metastructure with magnetic and conductive polymer layers enables broadband, low-reflection, and ultra-high absorption shielding in the sub-terahertz regime, positioning this research at an advanced, near-frontier level within the field of electromagnetic radiation shielding.

## Conclusions

We have designed and characterized flexible multilayer composites for electromagnetic radiation (EMR) shielding in the *90-330* GHz band by combining conductive carbon fabric with magnetic polymer/inorganic coatings. The multilayer composition was selected using a functionally graded design strategy, illustrating how impedance matching and combined loss mechanisms may be achieved in thin, flexible composites without reliance on extensive empirical optimization. The carbon fabric substrate featured a regular twill (diagonal) metastructure, while sequential coatings of epoxy filled with graphite, Ni, or Fe$_2$O$_3$ introduced additional conductive and magnetic loss channels. This hybrid design yielded thin (*∼ 1-2* mm), mechanically flexible composites with absorption-dominant shielding performance.

**Table 1:** Characteristic parameters of the components of the electromagnetic radiation shielding composite.

Sample	Fabric	Layer #1			Layer #2				Layer #3			Composite
	$a_0$	Epoxy	GNP6	$a_1$	Epoxy	Ni	*γ*-Fe$_2$O$_3$	$a_2$	Epoxy	GNP6	$a_3$	*a*
	mm	%	%	mm	%	%	%	mm	%	%	mm	mm
Main Samples												
#1	1.0	100	−	0.3	−	−	−	−	−	−	−	1.3
#2	1.0	94	6	0.3	68	32	−	0.3	−	−	−	1.6
#3	1.0	94	6	0.3	77	−	23	0.3	94	6	0.2	1.8
Auxiliary Samples												
#2$^\prime$	1.0	−	−	−	68	32	−	0.3	−	−	−	1.3
#3$^\prime$	1.0	−	−	−	77	−	23	0.3	94	6	0.2	1.5

**Table 2:** Benchmark comparison of representative electromagnetic shielding and absorbing materials.

Material system	Frequency range	Max. SE (dB)	Thickness	Dominant mechanism	Structural features	Key limitations	Ref.
Carbon black/polymer composites	MHz–10 GHz	20–40	2–5 mm	Reflection-dominated	Random conductive filler network	Poor impedance matching, narrowband	^7–10^
CNT/polymer composites	GHz	30–60	1–4 mm	Reflection + absorption	Percolated CNT networks	High reflection, cost, dispersion issues	^8,9^
Graphene aerogels	GHz	40–50	3–10 mm	Absorption-dominated	Ultralight porous 3D network	Fragile, relatively thick	^28^
Porous graphene/polymer foams	2–18 GHz	40–60	2–5 mm	Absorption-dominated	Hierarchical porous structure	Limited high-frequency performance	^29^
Ferrite/polymer absorbers (Fe$_3$O$_4$, NiZn)	1–30 GHz	30–50	2–6 mm	Magnetic loss	Magnetic resonance fillers	Narrowband, high density	^13,16,17^
Carbon–magnetic hybrid foams	GHz	40–60	2–4 mm	Dielectric + magnetic loss	3D porous magnetic networks	Scarce sub-THz validation	^33,36^
Planar metamaterial absorbers	Narrowband (GHz–THz)	20–40	*<1* mm	Resonant absorption	Lithographically patterned unit cells	Narrowband, rigid, complex fabrication	^42,43^
Plasmonic Ti$_3$C$_2$T$_x$ MXene metamaterial absorber	Visible–NIR	Broadband optical absorption	Thin film	Localized plasmonic absorption	Nanostructured MXene metamaterial supporting surface plasmon resonances	Complex nanofabrication; not designed for EMI shielding applications	^60^
Hybrid metamaterial–absorber stacks	GHz	40–60	1–3 mm	Multi-resonant absorption	Patterned + lossy multilayers	Fabrication complexity	^43^
Aligned compact Ti$_3$C$_2$T$_x$ MXene film	0.2–1.6 THz (sub-THz)	59	2.25 *μ*m	Reflection + absorption	Centrifugal-sprayed aligned nanosheets	MXene oxidation under humidity	^61^
Carbon fabric + magnetic multilayer (this work)	90–330 GHz	75–80	*∼*1–2 mm	Absorption-dominated	Woven metastructure + magnetic and conductive layers	No intrinsic limitations identified	This work

Measurements confirmed ultra-low reflection (*<5* dB above 250 GHz) and exceptionally high absorption, with shielding efficiency reaching up to *∼ 80* dB (*>99.99* % attenuation). The results demonstrate that (i) the carbon fabric metastructure governs the angular and polarization dependence of the response, (ii) magnetic layers broaden the absorption bandwidth through additional magnetic-loss-assisted dissipation and improved impedance matching, and (iii) the combined incorporation of conductive and magnetic coatings enhances absorption and suppresses reflection through coupled dielectric, magnetic, and geometric interactions within the multilayer architecture. The results further indicate that the absorption performance is governed by the compositional grading and sequence of conductive and magnetic layers, which jointly control impedance matching, internal reflections, and the coupling of dielectric and magnetic losses.

The combined interaction between the carbon fabric metastructure and magneto-responsive coatings provides a versatile strategy for tailoring impedance matching, resonance conditions, and broadband absorption. The developed composites exhibit promising potential for applications in radar stealth, 6G/terahertz communications, and medical shielding, where ultrahigh absorption and minimal backscattering are crucial.

## Supplementary Information

Below is the link to the electronic supplementary material: detailed measurement results and compositional data for the samples discussed in the main manuscript.

## Supplementary Information

Supplementary Information.

## Data Availability

The data that support the findings of this study are available from the corresponding author upon reasonable request.
