# Supplementary material for: Synergistic effect of carbon fabric metastructure and magnetic layers in a composite for electromagnetic radiation shielding
Source: Sci Rep. 2026 Jul 8;16:31429. doi: 10.1038/s41598-026-61026-3 (PMC13645620; doi:10.1038/s41598-026-61026-3)
Supplement: Supplementary file 1 [file 41598_2026_61026_MOESM1_ESM.pdf]

SUPPLEMENTARY INFORMATION

Synergistic effect of carbon fabric metastructure and magnetic layers in a composite for electromagnetic radiation shielding

Y. Yashchyshyn, A. Zaichenko, Kh. Harhay, N. Mitina, K. Godziszewski, O. Izhyk,
I. Demydov, R. Nebesnyi, V. Khardikov, V. Tuz

This document provides detailed measurement results and compositional data for the samples discussed in the main manuscript. The specific material configurations are defined in Table S1, while their corresponding absorption and reflection characteristics across the 90-330 GHz range are compiled in Figs. S1-S6.

The investigation into these multilayered composites reveals a distinct hierarchy in shielding effectiveness (SE) based on material composition. Notably, samples incorporating nickel-coated carbon fillers (specifically ED_2 and ED_2-Ni), demonstrate significantly superior shielding properties compared to those utilizing iron oxide (ED_2-Fe_2O_3). This enhancement is attributed to the high conductivity and magnetic permeability of the nickel components, which provide a more robust barrier against high-frequency radiation.

A critical finding of this study is the evidence of structural synergy within the multilayered architectures. The base sample, ED_0, establishes the fundamental electromagnetic distribution, which is largely dictated by the periodic metastructure of the carbon fabric. However, the addition of subsequent layers enhances performance non-linearly. This is most evident when comparing the 4-layer ED_3 configuration to the 3-layer ED_2. The ED_3 sample does not merely aggregate the shielding values of its individual components; instead, the interaction between the graphite nanoplatelet (GNP6) layers and the magnetic core creates a synergistic effect that optimizes energy dissipation.

Analysis of the physical shielding mechanisms allows for a clear distinction between surface reflection and internal loss. It was observed that reflection (SE_R) remains consistently low and nearly identical across all samples, regardless of filler type or layer count. This indicates that the high total shielding effectiveness is not a result of surface-level reflection, but is driven almost entirely by internal absorption (SE_A) and multiple scattering events. The woven geometry of the carbon fabric acts as a scattering center, effectively trapping electromagnetic waves within the composite where they are subsequently attenuated by the dielectric and magnetic losses of the filler particles.

Table S1. Characteristic parameters of the components of the electromagnetic radiation shielding composite.

Sample #	Title	Fabric thickness a_0, mm	Composition										Composite thickness a, mm
			Layer #1			Layer #2				Layer #3			
							Magnetic particles						
			Epoxy %	GNP6 %	a_1, mm	Epoxy %	Ni %	γFe_2O_3 %	a_2, mm	Epoxy %	GNP6 %	a_3, mm	
**1**	**ED_0**	**1.0±0.1**	**100**	**-**	**0.3±0.1**	**-**	**-**	**-**	**-**	**-**	**-**	**-**	**1.3±0.1**
	ED_1	1.0±0.1	74	6	0.3±0.1	-	-	-	-	-	-	-	1.3±0.1
**2**	**ED_2**	**1.0±0.1**	**94**	**6**	**0.3±0.1**	**68**	**32**	**-**	**0.5±0.1**	**-**	**-**	**-**	**1.8±0.1**
	ED_2-Ni	1.0±0.1	94	6	0.3±0.1	68	32	-	0.5±0.1	94	6	0.3±0.1	2.1±0.1
	ED_2-Fe_2O_3	1.0±0.1	74	6	0.3±0.1	77	-	23	0.5±0.1	-	-	-	1.8±0.1
**3**	**ED_3**	**1.0±0.1**	**94**	**6**	**0.3±0.1**	**77**	**-**	**23**	**0.5±0.1**	**94**	**6**	**0.3±0.1**	**2.1±0.1**

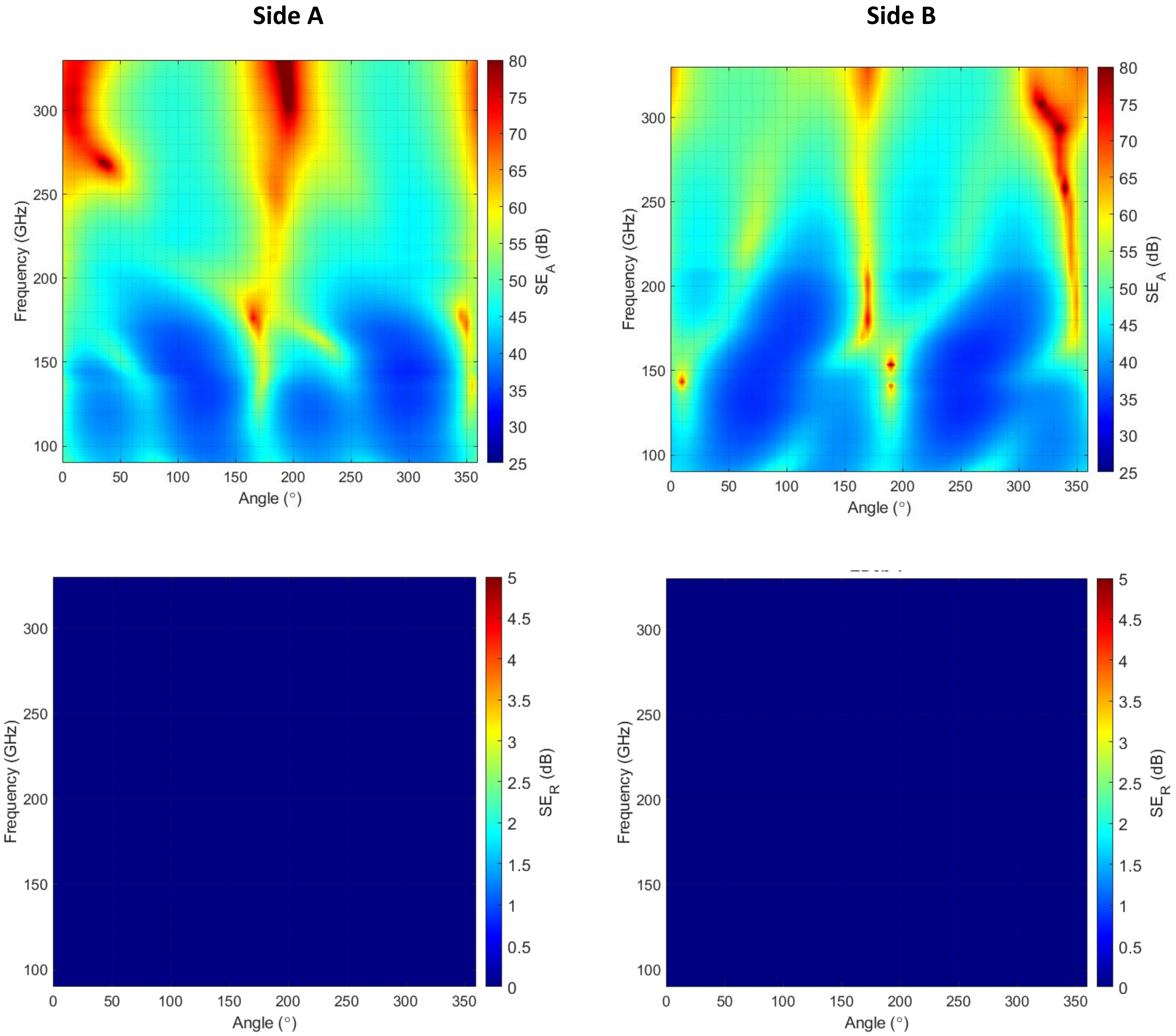

Fig. S1. Absorption (SE_A) and reflection (SE_R) shielding efficiencies of composite ED_0. Left and right panels and show efficiencies for irradiation from Side A and Side B, respectively. Efficiencies are plotted as functions of polarization angle (horizontal axis) and frequency (vertical axis). Color scales indicate efficiency in decibels (dB).

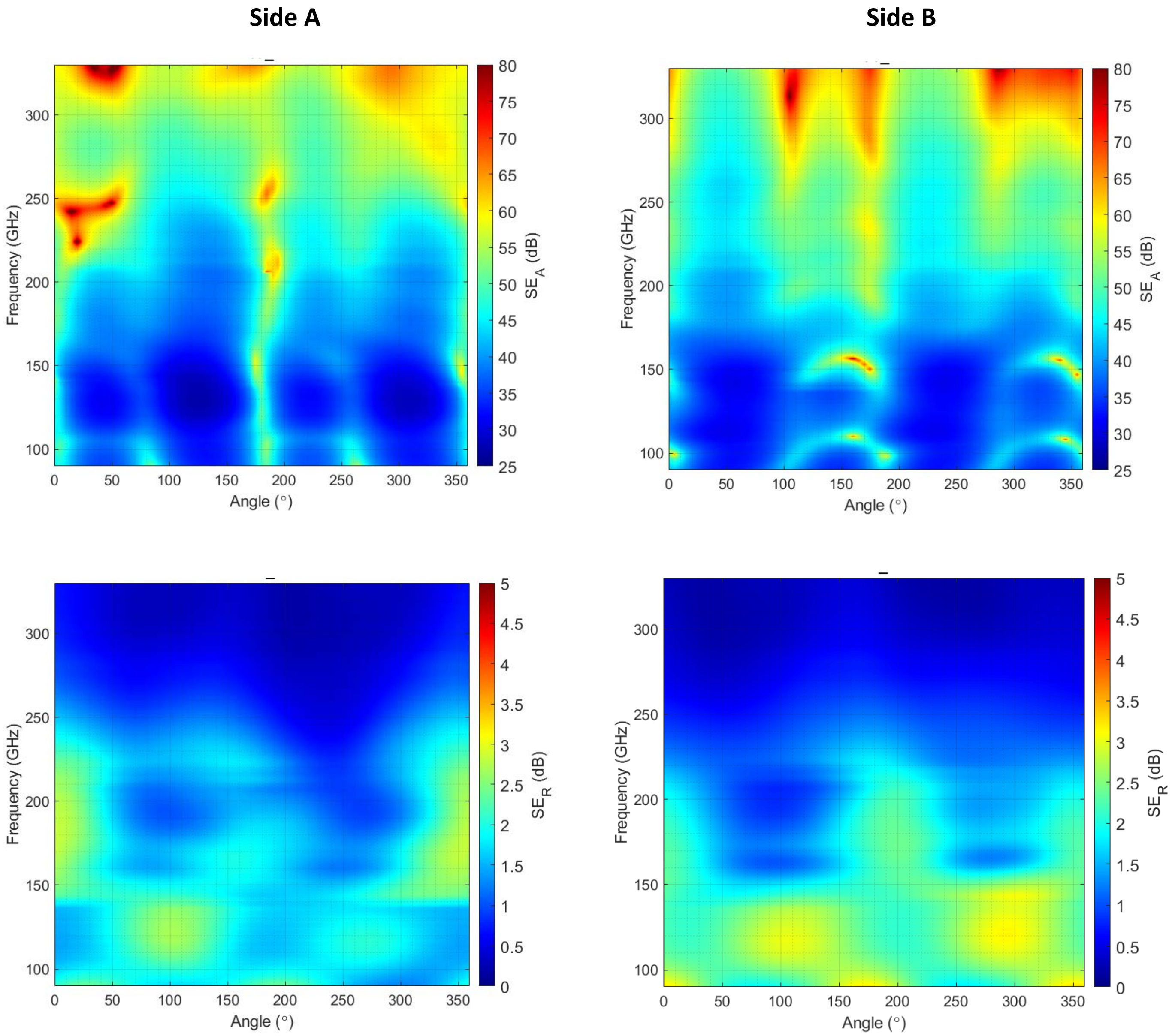

Fig. S2. Absorption (SE_A) and reflection (SE_R) shielding efficiencies of composite ED_1. Left and right panels and show efficiencies for irradiation from Side A and Side B, respectively. Efficiencies are plotted as functions of polarization angle (horizontal axis) and frequency (vertical axis). Color scales indicate efficiency in decibels (dB).

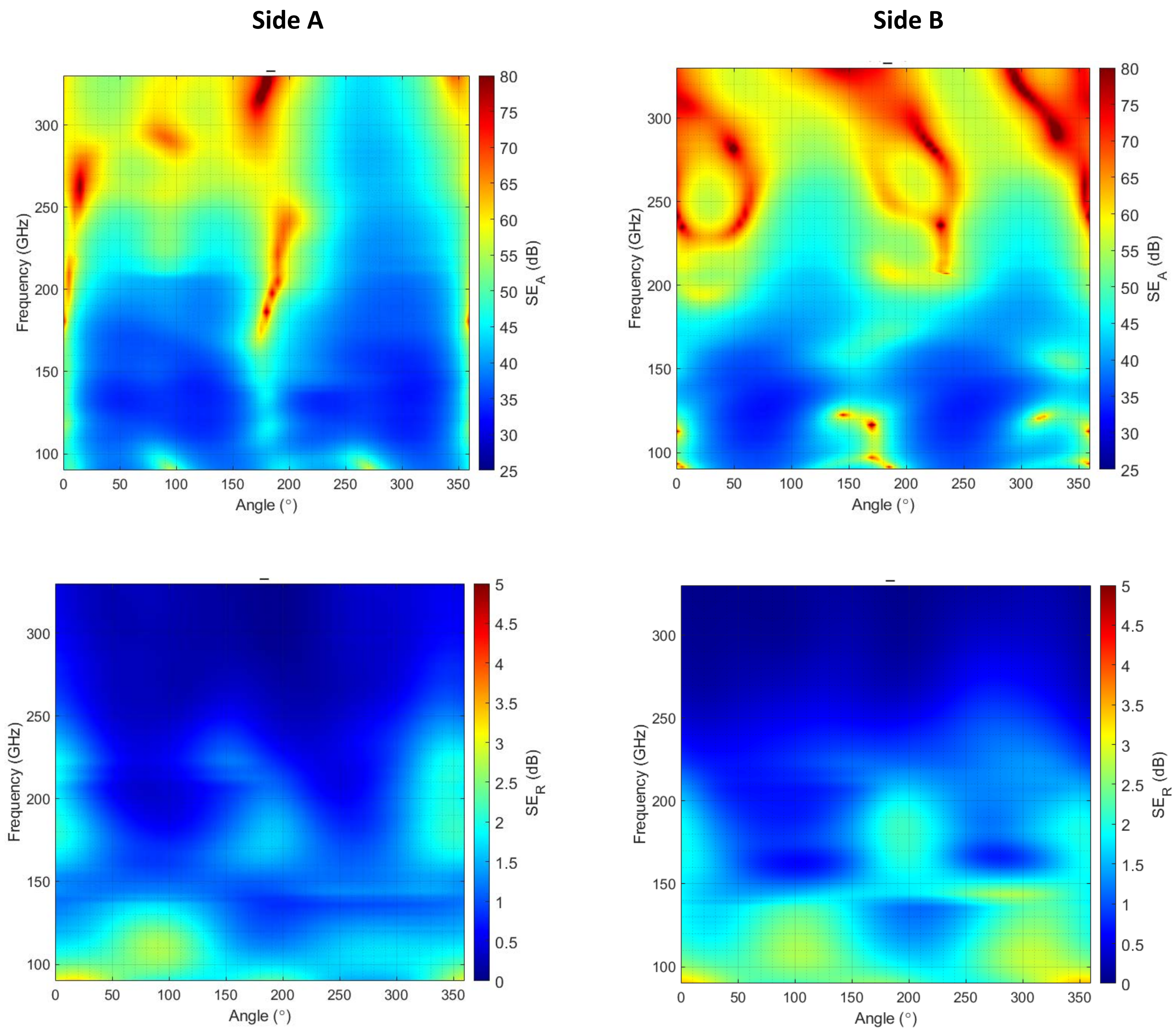

Fig. S3. Absorption (SE_A) and reflection (SE_R) shielding efficiencies of composite ED_2. Left and right panels and show efficiencies for irradiation from Side A and Side B, respectively. Efficiencies are plotted as functions of polarization angle (horizontal axis) and frequency (vertical axis). Color scales indicate efficiency in decibels (dB).

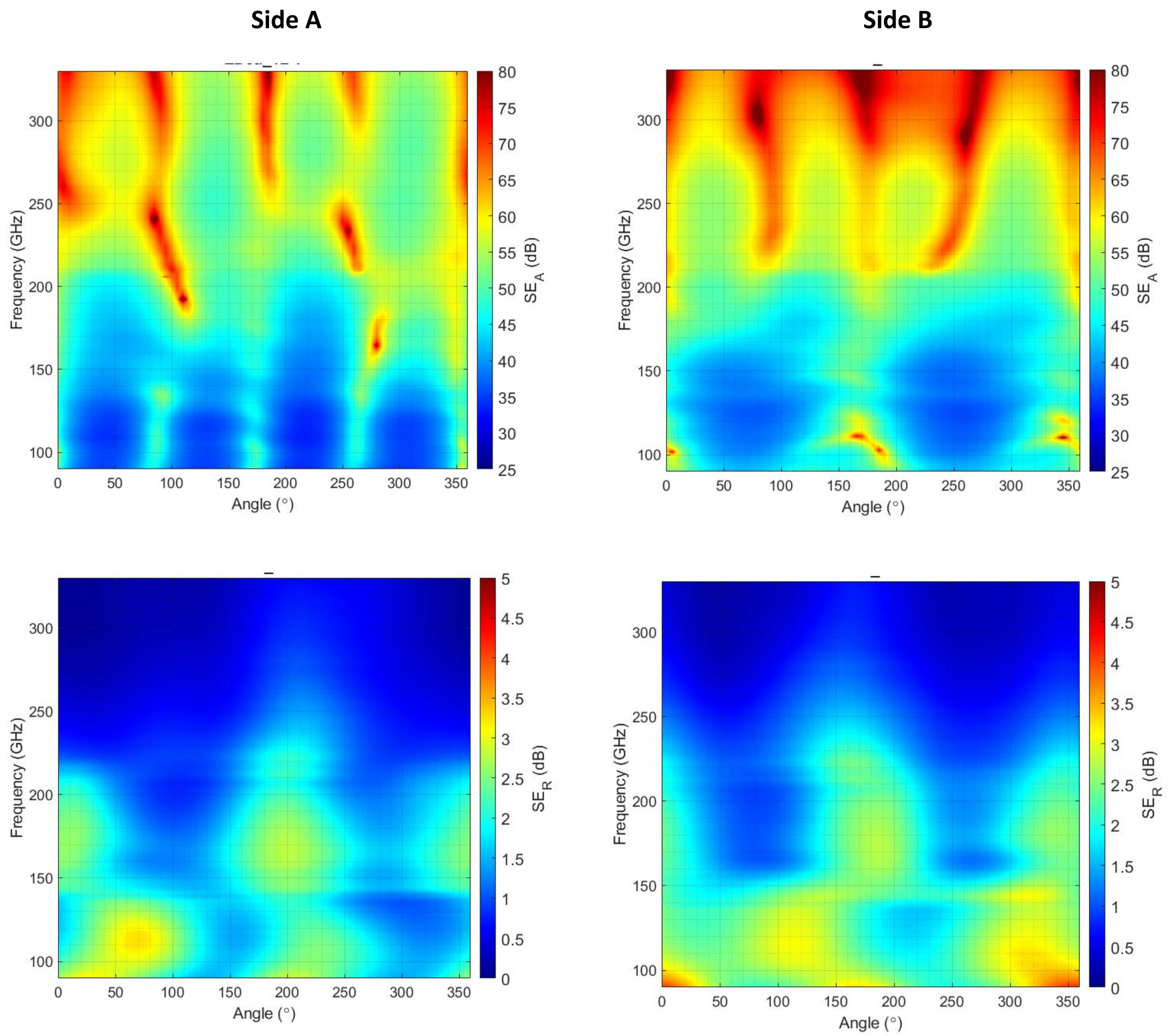

Fig. S4. Absorption (SE_A) and reflection (SE_R) shielding efficiencies of composite ED_2-Ni. Left and right panels and show efficiencies for irradiation from Side A and Side B, respectively. Efficiencies are plotted as functions of polarization angle (horizontal axis) and frequency (vertical axis). Color scales indicate efficiency in decibels (dB).

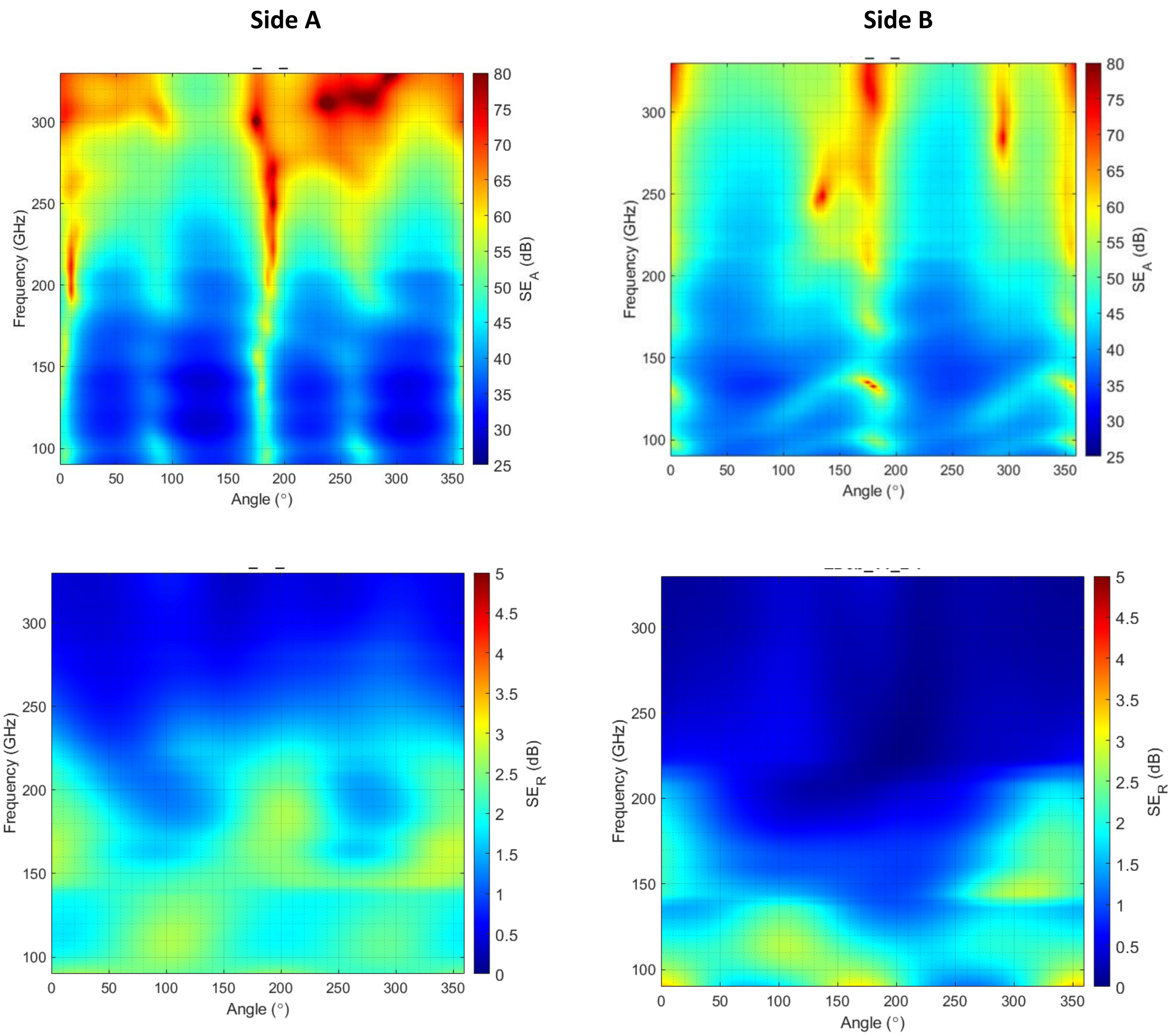

Fig. S5. Absorption (SE_A) and reflection (SE_R) shielding efficiencies of composite ED_2-Fe_2O_3. Left and right panels and show efficiencies for irradiation from Side A and Side B, respectively. Efficiencies are plotted as functions of polarization angle (horizontal axis) and frequency (vertical axis). Color scales indicate efficiency in decibels (dB).

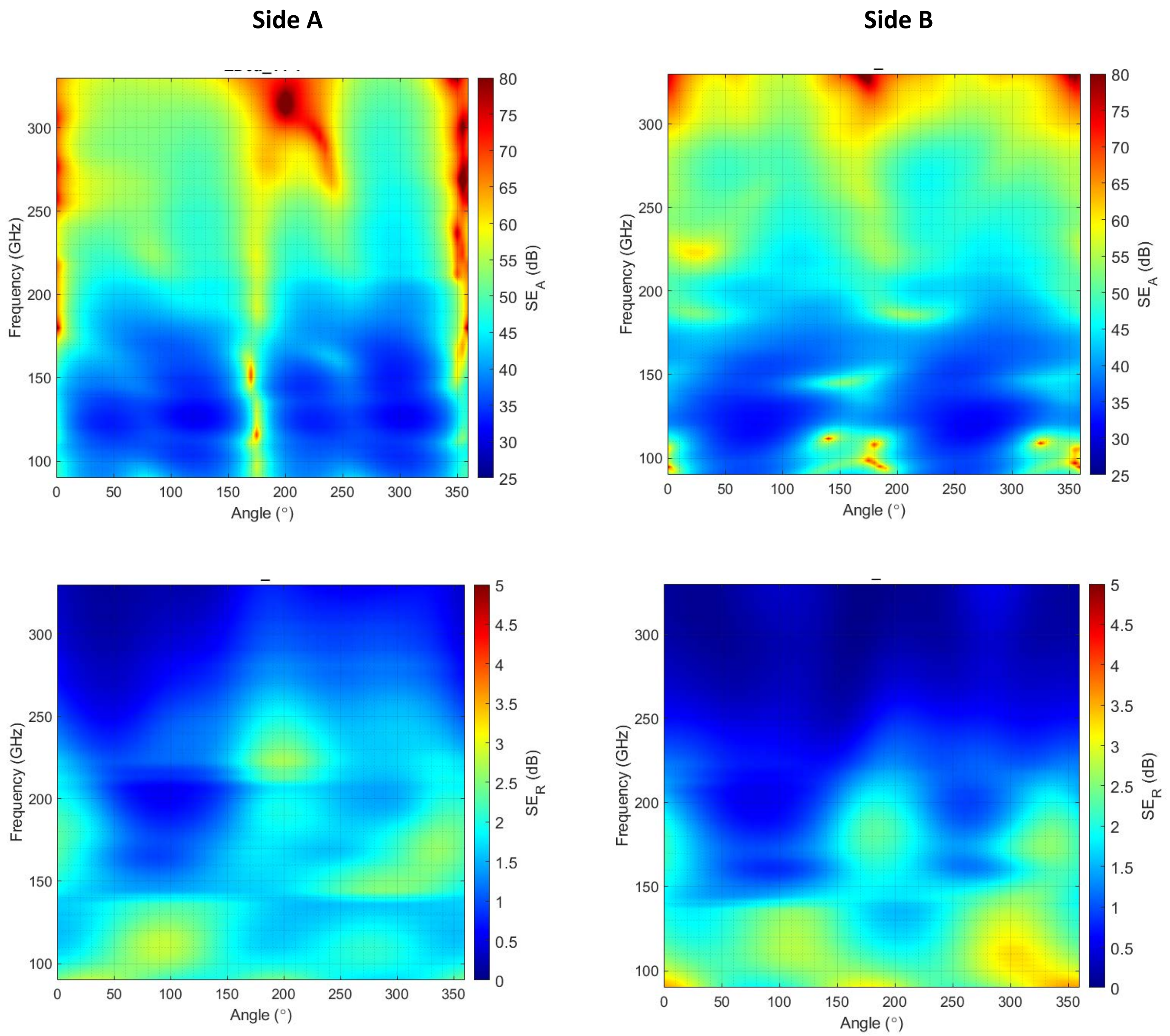

Fig. S6. Absorption (SE_A) and reflection (SE_R) shielding efficiencies of composite ED_3. Left and right panels and show efficiencies for irradiation from Side A and Side B, respectively. Efficiencies are plotted as functions of polarization angle (horizontal axis) and frequency (vertical axis). Color scales indicate efficiency in decibels (dB).
